# Single molecule MATAC-seq reveals key determinants of DNA replication origin efficiency

**DOI:** 10.1093/nar/gkad1022

**Published:** 2023-11-13

**Authors:** Anna Chanou, Matthias Weiβ, Karoline Holler, Atiqa Sajid, Tobias Straub, Jana Krietsch, Andrea Sanchi, Henning Ummethum, Clare S K Lee, Elisabeth Kruse, Manuel Trauner, Marcel Werner, Maxime Lalonde, Massimo Lopes, Antonio Scialdone, Stephan Hamperl

**Affiliations:** Institute of Epigenetics and Stem Cells, Helmholtz Zentrum München, Munich, Germany; Institute of Epigenetics and Stem Cells, Helmholtz Zentrum München, Munich, Germany; Institute of Epigenetics and Stem Cells, Helmholtz Zentrum München, Munich, Germany; Institute of Functional Epigenetics, Helmholtz Zentrum München, Neuherberg, Germany; Institute of Computational Biology, Helmholtz Zentrum München, Neuherberg, Germany; Institute of Epigenetics and Stem Cells, Helmholtz Zentrum München, Munich, Germany; Core Facility Bioinformatics, Biomedical Center, Faculty of Medicine, Ludwig-Maximilians-Universität München, Martinsried, Germany; Institute of Molecular Cancer Research, University of Zurich, Zurich, Switzerland; Institute of Molecular Cancer Research, University of Zurich, Zurich, Switzerland; Institute of Epigenetics and Stem Cells, Helmholtz Zentrum München, Munich, Germany; Institute of Epigenetics and Stem Cells, Helmholtz Zentrum München, Munich, Germany; Institute of Epigenetics and Stem Cells, Helmholtz Zentrum München, Munich, Germany; Institute of Epigenetics and Stem Cells, Helmholtz Zentrum München, Munich, Germany; Institute of Epigenetics and Stem Cells, Helmholtz Zentrum München, Munich, Germany; Institute of Epigenetics and Stem Cells, Helmholtz Zentrum München, Munich, Germany; Institute of Molecular Cancer Research, University of Zurich, Zurich, Switzerland; Institute of Epigenetics and Stem Cells, Helmholtz Zentrum München, Munich, Germany; Institute of Functional Epigenetics, Helmholtz Zentrum München, Neuherberg, Germany; Institute of Computational Biology, Helmholtz Zentrum München, Neuherberg, Germany; Institute of Epigenetics and Stem Cells, Helmholtz Zentrum München, Munich, Germany

## Abstract

Stochastic origin activation gives rise to significant cell-to-cell variability in the pattern of genome replication. The molecular basis for heterogeneity in efficiency and timing of individual origins is a long-standing question. Here, we developed Methylation Accessibility of TArgeted Chromatin domain Sequencing (MATAC-Seq) to determine single-molecule chromatin accessibility of four specific genomic loci. MATAC-Seq relies on preferential modification of accessible DNA by methyltransferases combined with Nanopore-Sequencing for direct readout of methylated DNA-bases. Applying MATAC-Seq to selected early-efficient and late-inefficient yeast replication origins revealed large heterogeneity of chromatin states. Disruption of INO80 or ISW2 chromatin remodeling complexes leads to changes at individual nucleosomal positions that correlate with changes in their replication efficiency. We found a chromatin state with an accessible nucleosome-free region in combination with well-positioned +1 and +2 nucleosomes as a strong predictor for efficient origin activation. Thus, MATAC-Seq identifies the large spectrum of alternative chromatin states that co-exist on a given locus previously masked in population-based experiments and provides a mechanistic basis for origin activation heterogeneity during eukaryotic DNA replication. Consequently, our single-molecule chromatin accessibility assay will be ideal to define single-molecule heterogeneity across many fundamental biological processes such as transcription, replication, or DNA repair *in vitro* and *ex vivo*.

## Graphical Abstract

**Graphical Abstract:**
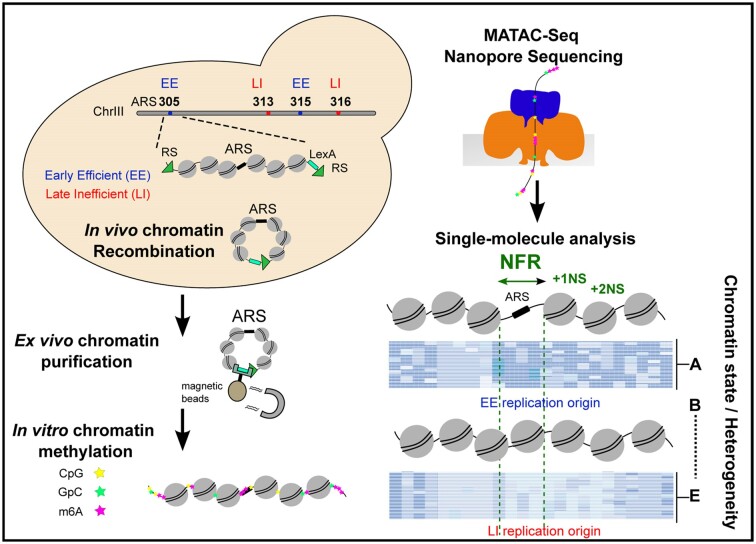

## Introduction

In eukaryotic genomes, multiple start sites of DNA replication named origins are distributed across each of the linear chromosomes. The assembly of replication forks and the bidirectional initiation of DNA synthesis at these sites is known as origin firing. The budding yeast *Saccharomyces cerevisiae* has been a particularly useful model organism to study eukaryotic replication, owing to the presence of short (∼200 bp) Autonomous Replication Sequences (ARS) that support the propagation and maintenance of plasmids and therefore define chromosomal replication origins (1,2). At the core of every yeast replication origin is a replicator sequence containing a conserved AT-rich 11bp stretch known as the ARS consensus sequence (ACS), which is essential for binding of the initiator complex known as the origin recognition complex (ORC). This six-subunit complex (Orc1-6) is highly conserved among eukaryotes and works in concert with Cdc6 and Cdt1 to direct the loading of the replicative helicase Mcm2–7 onto DNA to form the pre-replicative complex (pre-RC). The assembly of the pre-RC in G1 serves to ‘license’ an excess of origins for activation in the subsequent S phase. Cyclin- and Dbf4-dependent kinase activities during S phase result in the recruitment of additional proteins at a fraction of loaded pre-RCs to form the preinitiation complex, to unwind the DNA, and produce bidirectional replication forks (3).

**Figure 1.**
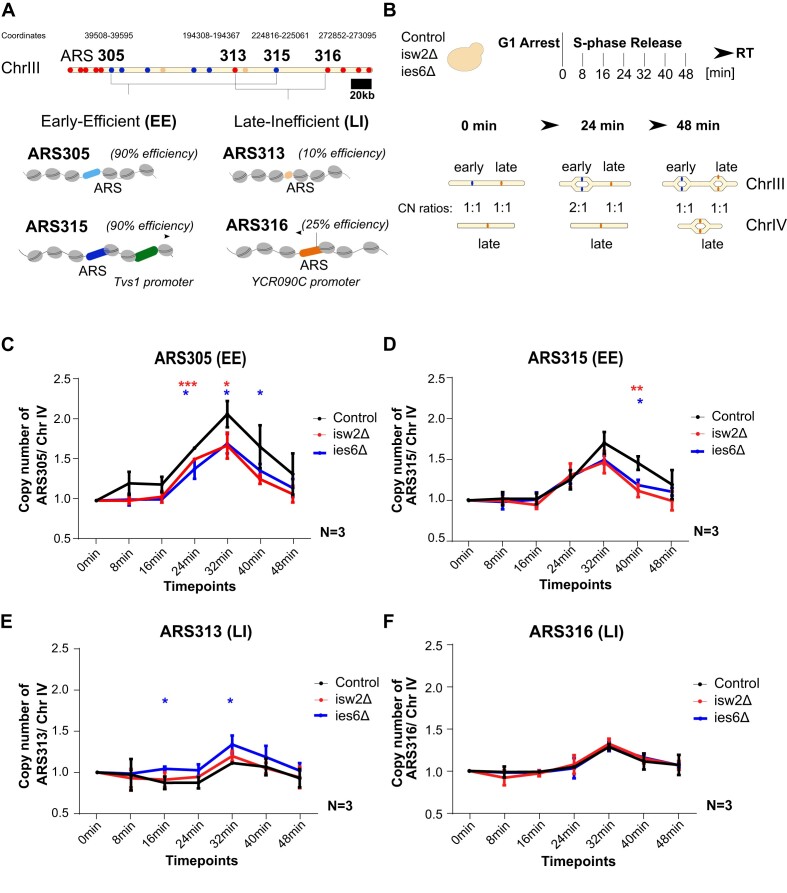
(**A**) Schematic representation of early- (blue), mid- (orange) and late- (red) replication origins on Chromosome III of *Saccharomyces cerevisiae*. The chromosomal coordinates of the four origins used in this study is indicated on the top. Scale bar = 20kb. The two Early-Efficient (EE) origins (ARS305/ARS315 – ARS blue) show 90% efficiency and two Late-Inefficient (LI) origins (ARS313/ARS316 – ARS orange) show 10% and 25% efficiency, respectively. Distinct chromatin features are indicated around the ARS loci such as nucleosomes (grey) and gene promoters (green). (**B**) Experimental outline for measuring replication timing of each origin (Control, isw2Δ, ies6Δ). After G1-arrest, the cells were released into S-phase and samples were collected every 8 min for FACS (Supplementary Figure S1C) and qPCR. A late replicating region of ChrIV is used as reference. The ratio between the origins of interest on ChrIII and the reference region shows the DNA copy number changes due to sequential duplication of the target and reference regions. (**C**–**F**) The replication timing plots show the average copy number ratios of ARS305, ARS315, ARS313, ARS316 compared to the late replicating region of ChrIV with standard deviation from *n* = 3 biological replicates (*, **, *** indicates statistical significance *P* < 0.05, or *P* < 0.01, or *P* < 0.001, respectively, unpaired *t*-test).

Similar to all other eukaryotic genomes, yeast replication origins can be classified according to two main intrinsic features, replication timing and efficiency. Replication timing denotes the timepoint when an origin fires and can occur at a continuum from early to late S phase (4,5). Replication efficiency describes the probability of activation for each replication origin (i.e. the fraction of cells in which the origin fires) and is defined as a combination of an origin's firing probability and its proximity to other origins that fire earlier and can therefore passively replicate the origin. Origin efficiencies display a wide range from inefficient (≤ 10%) to highly efficient (≥90%) (6) (Figure 1A). The difference in replication timing and efficiency of individual origins scattered across the chromosomes defines the replication timing program that shows a highly reproducible order of chromosomal replication at the population level. However, at the single cell level, origin firing is an intrinsically stochastic event with no two chromosomes exhibiting the same replication pattern in an isogenic cell population (7,8). This apparent contradiction can be explained by the fact that the population experiments average the behavior of individual origins over large numbers of cells, obscuring stochastic effects. The observed stochasticity of origin activation eventually leads to further cell-to-cell variability (6,9–15). Thus, the molecular rules based on which individual cells choose to activate a specific, highly individualized subset of replication origins remains an outstanding fundamental question (9,16).

A plausible mechanism by which firing probability could be regulated is the local chromatin structure. Yeast ARS sequence is AT-rich which is inhibitory to nucleosome assembly and forms a nucleosome-free region (NFR). An NFR at an ARS provides an accessible environment for ORC binding and replication initiation (17,18). Importantly, ORC-binding at functional ARS improves nucleosomal positioning of the −1 and +1 nucleosomes surrounding the ARS. Disruption of this ORC-directed nucleosome phasing by mutating the ORC binding site or moving one nucleosome further from the ARS interferes with the efficiency of origin firing (18,19). Genome-wide nucleosome positioning maps in G1-phase revealed many different clusters of distinct nucleosome occupancy patterns around annotated origins (20), suggesting large heterogeneity of different chromatin states. A later study showed that early-origins tend to show higher nucleosome occupancy at the +1 and −1 position around the ARS sites compared to late origins (21), suggesting that the interaction of ORC with origin DNA and neighboring nucleosomes is critical for efficient and timely replication. This is also consistent with recent *in vitro* reconstitution experiments (22) that show stable ORC-nucleosome complexes that can efficiently load the MCM2-7 double hexamer onto adjacent nucleosome-free DNA. Neighboring genomic features, most notably transcription start sites (TSS) and gene ends, were also shown to influence the nucleosome positioning and thus the replication identity of an origin (20). Together, these data suggest a model of nucleosome positioning at replication origins in which the underlying ARS sequence occludes nucleosomes to permit binding of ORC, which then likely in concert with nucleosome modifiers or chromatin remodeling enzymes (CREs) position nucleosomes at origins in a favorable way to promote replication origin function. In fact, a recent study showed that ORC plays an active role in establishing the chromatin architecture of yeast replication origins (23). By genome-wide biochemical reconstitution assays, it was shown that the Orc1 subunit in collaboration with the redundant chromatin remodelers INOsitol-requiring 80 (INO80), Imitation SWitch subfamily (ISW1a), ISW2 and Chromatin organization modifier, helicase and DNA-binding domains (Chd1) establishes the NFR as well as flanking nucleosomal arrays at yeast origins.

Because CREs are major architects of chromatin structure (24–33), we hypothesized that comparing chromatin accessibilities in wildtype and CRE mutant strains at selected replication origins will allow us to extract the key differences in chromatin states that are important for the functional activation of DNA replication origins. However, critical testing of this hypothesis necessitates the analysis of origin chromatin structure at the level of single cells or single DNA molecules. Long-read nanopore sequencing methods have recently come into focus for the study of replication dynamics in both yeast and mammalian genomes by the detection of the thymidine analogue bromodeoxyuridine after short pulse-labeling in asynchronously growing cells (34–36). This allowed accurate mapping of early S phase origins, and reconstruction of a genome-wide map of fork progression and fork stalling events based on individual single-molecule fork rates (35–37). However, a single-molecule method to profile the underlying chromatin accessibility in the context of replication origin sites is still missing. Therefore, to potentially determine the precise nucleosome configuration(s) restricting or allowing replication initiation, we developed Methylation Accessibility of TArgeted Chromatin domain Sequencing (MATAC-Seq) to define single-molecule chromatin accessibility maps of specific genomic loci after targeted purification in their native chromatin context. Applying MATAC-Seq to selected early-efficient (EE) and late-inefficient (LI) budding yeast replication origins revealed large cell-to-cell heterogeneity in their chromatin states. Disruption of INO80 or ISW2 chromatin remodeling complexes leads to changes at individual nucleosomal positions that correlate with changes in their replication efficiency. Thus, MATAC-Seq identifies the large spectrum of alternative nucleosome occupancy states that co-exist on a given locus. In replication origins, this allowed us to extract key chromatin features of stochastic origin activation previously masked in common population-based techniques and provides a mechanistic basis for origin activation heterogeneity during DNA replication of eukaryotic cells.

## Materials and methods

Unless noted otherwise, standard molecular biology techniques were used for cloning of plasmids and lithium acetate transformation of yeast cells. Complete list of yeast strains with genotypes can be found in Supplementary Table S1. Complete list of oligonucleotides can be found in Supplementary Table S2. Complete list of plasmids can be found in Supplementary Table S3.

### Spot test

Yeast strains were grown overnight in 5 ml of YPD medium at 30°C and 200 rpm. After measuring the optical density of each strain and dilution to OD_600_ 1.0, five serial 1/10 dilutions were performed and 7 μl of medium containing the diluted yeast cells were transferred on YPD agar plates or YPD agar plates containing 0,05% MMS or 100 mM HU and incubated at 30°C for a maximum of 3 days. The colony size of the yeast strains was monitored daily.

### Culture of yeast strains expressing the recombination cassette

Yeast strains competent for recombination were inoculated to OD_600_ 0.2 in 2 l YPR medium at 30°C and 200 rpm. At OD_600_ 1.0, cells were arrested in G1-phase by α-factor (50 ng/ml) and, simultaneously, recombination was induced by addition of galactose to a final concentration of 2% (w/v) for 2 h at 30°C. Cells were harvested by centrifugation (6000 × g, 10 min, 4°C), the supernatant was discarded, and the cell pellets resuspended with water and collected in a sealed 25 ml syringe. After pelleting the cells (3000 × g, 12 min, 4 min) and discarding the supernatant, the cells were extruded into liquid nitrogen, broken in small ‘spaghetti’ pieces and stored at −80°C for later use.

### Replication timing

Yeast cells were inoculated at OD_600_ 0.2 in 2 l YPD medium at 30°C and 200 rpm. At OD_600_ 0.6, cells were arrested in G1-phase by α-factor (50 ng/ml) for 2 h. Samples of 1 ml culture for flow cytometry analysis were obtained every 30 min (0, 30, 60, 90, 120 min). After 2 h G1-arrest, cells were released into S-phase by addition of 125 U of Pronase (Sigma-Aldrich, 53702-25KU) and potassium phosphate buffer to final concentration of 20 mM. Samples for flow cytometry and quantitative PCR (1 and 4.5 ml, respectively) were collected every 8 min for 48 min and processed separately according to the methods below.

### Replication timing: *quantitative PCR*

The samples were supplemented with 500 μl of 1% sodium azide solution (w/v) in 0.2 M EDTA, washed once with water (3000 × g, 3 min, 4°C) and the resulting yeast pellets were snap frozen in liquid nitrogen. For DNA extraction, the pellets were resuspended into RINB (50 mM Tris–HCl pH 8, 0.1 M EDTA, 0.1% (v/v) beta mercaptoethanol) with Zymolyase to a final concentration of 2% (w/v) and were incubated for 1 h at 37°C and 700 rpm shaking. After addition of 2 μl RNAse A (10mg/ml), spheroblasts were incubated for 1 h at 37°C, followed by the addition of 5 μl Proteinase K (10mg/ml) and 10 μl SDS (20%) at 55°C for 1 h. DNA was isolated by phenol-chloroform extraction (1:1) followed by overnight ethanol precipitation. DNA pellets were suspended in 50 μl of H_2_O and 10 μg of DNA was digested by EcoRI restriction endonuclease (NEB) at 37°C for 1 h. DNA samples were diluted 1:10 with H_2_O and analyzed by qPCR using SybrGreen mastermix (BioRad). In each of the reaction wells, the same qPCR master mix was used for one of the origin loci (ARS305, ARS315, ARS316 or ARS313) using primer pairs #0463/#0464, #1035/#1036, #0837/#0838 and #0552/#0553, respectively and the late replicating region from ChrIV, ChrV or ChrXIV using primer pairs #0834/#0835, #1299/#1300 or #1301/#1302, respectively. The copy number ratio of each locus to ChrIV was normalized to the G1-arrested sample (0 min timepoint) and set to 1. For each qPCR reaction, 5 technical replicates of each timepoint were performed with *n* = 3 biological replicates.

### Replication timing: *flow cytometry*

Cells were centrifuged at 16 000 × g for 2 min. The supernatant was discarded and 1 ml of cold 70% ethanol was added slowly drop-by-drop with gentle agitation. The fixed cell suspensions were stored at 4°C until further use. 500–600 μl of the samples were transferred into a clean 1.5 ml reaction tube, centrifuged and the resulting pellets resuspended with 300 μl 50 mM Na-citrate and 0.1mg/ml RNAseA. After 2 h incubation at 50°C, 3 μl Proteinase K (10 mg/ml) was added, followed by another 2 h incubation at 50°C. 30 μl of each sample were mixed with 170 μl of 50 mM Na-citrate and 0.5 μM Sytox Green (S7020, ThermoFisher). Prior to the FACS analysis, the samples were briefly sonicated (Bioruptor, 5min with 30 s ON and OFF intervalls) to detach cell clumps before proceeding with the flow cytometry analysis on a BD FACSCanto™ device.

### Restriction enzyme accessibility assay of linear, recombined and affinity-purified chromatin

Cells were grown to OD_600_ 0.8 in 50 ml of YPR medium at 30°C and 200 rpm. 25 ml of yeast culture were centrifuged (3000 × g, 10 min, 4°C), washed twice with H_2_O, snapfrozen in liquid nitrogen and were stored at −20°C for later use. Cell pellets were washed three times with Buffer A (15 mM Tris–HCl pH 7.4, 0.2 mM Spermine, 0.5 mM Spermidine, 80 mM KCI) and 1× Protease Inhibitors (16 rpm, 2 min, 4°C) and then resuspended in 350 μl of Buffer A × Protease Inhibitors and ∼450 μl glass beads (1mm, BioSpec Products). The buffer solution should be enough to cover the beads by a thin layer. The cell pellets were vortexed thoroughly for 2 × 10 min with interval break of 5 min. To collect the cell lysates, the bottom and lid of microtubes were pierced with a hot needle and placed in a 15ml tube. After centrifugation (130 × g, 1 min at 4°C), the glass beads remained in the microtubes. The crude cell lysates, collected in the 15ml tubes, were transferred into new 1.5ml microtubes. The 350 μl suspension was supplemented with 2 mM MgCl_2_ to a total volume of 400 μl. To achieve restriction digestion of chromatin loci by the indicated restriction enzymes, the crude nuclei or purified chromatin rings, were split into different reaction tubes for each restriction enzyme tested. Digestion was performed for 60 min at the optimal reaction temperature using different amounts of the respective restriction endonuclease (10 or 50 U and 50 or 100 U as indicated). The reaction was terminated by adding IRN buffer (50mM Tris–HCl at pH 8, 20 mM EDTA, 0.5 M NaCl) in a 1:1 ratio. Samples were treated with 2 μl RNAse A (10mg/ml) followed by 1 h incubation at 37°C and 5 μl Proteinase K (10 mg/ml) followed by 1 h incubation at 56°C. DNA was isolated by phenol-chloroform extraction (1:1) followed by overnight ethanol precipitation. DNA was linearized by restriction enzyme digestion overnight at 37°C in a final volume of 50 μl. The DNA samples were subjected to indirect end-labeling Southern blot analysis.

### Southern blot

Nucleic acids from genomic DNA were separated on a 1% agarose gel and blotted onto a positive Nylon membrane (Amersham Hybond™-N, GE) by capillary transfer in 1 M Ammonium acetate. DNA probes for hybridization were generated using the RadPrime DNA labeling system (Invitrogen) with incorporation of [α-32P] dATP (Hartmann Analytik) according to the instructions of the manufacturer. Images were acquired with Typhoon FLA 7000 imaging system.

### Specific chromatin domain isolation by affinity purification

A commercial coffee grinder (Gastroback, 42601) was pre-cooled by grinding 30 g of dry ice for 30sec. The resulting powder of dry ice was discarded. Appropriate amount of frozen cell pellets (3–4 g of multi copy ribosomal ARS (rARS) strain or 5–6 g of single copy ARS strains) were mixed with ∼60 g of dry ice and ground in the coffee mill. The coffee grinder is run for 10 × 30 s, with short intervals in between. Occasional tapping with a spatula against the outside of the mill prevents ground cells from sticking in a layer to the inside wall of the grinder. The fine powder of ground yeast was transferred into a plastic beaker and kept at RT until majority of dry ice has evaporated. For MATAC-Seq experiments, frozen pellets of each yeast strain were ground separately and then pooled together for subsequent affinity purification. After evaporation of dry ice, the powder is dissolved in 0.75 ml of cold buffer MB (20 mM Tris–HCl pH 8.0, 200 mM KCl, 5 mM MgAc, 0.5% Triton X-100 (w/v), 0.1% Tween-20 (w/v)) with 1× Protease and Phosphatase Inhibitors (Protease and Phosphatase Inhibitor Cocktail 100x, Thermo Fisher Scientific) per 1 g of ground yeast cells. The cell lysate was transferred into 2 ml low-binding reaction tubes (Eppendorf) and was cleared from cell debris by centrifugation for 30 min in a microcentrifuge at 16.000 × g and 4°C. Supernatant containing the diluted chromatin rings was transferred into 2 ml low-binding tubes and incubated with IgG-coupled magnetic beads (500 μl slurry of beads per 4 g of frozen cell pellet) (38). DNA and protein samples (0.1% and 0.05%, respectively) were obtained from the supernatant (Input IN) before the addition of magnetic beads. Supernatant was incubated with the beads for 2 h on a rotating wheel at 4°C. Beads were separated from the supernatant using a magnetic rack and samples for DNA and protein analysis were collected from the resulting Flow Through (FT) (0.1% and 0.05%, respectively). The beads were washed 5 times with 1 ml cold buffer MB with 1× Protease and Phosphatase inhibitors and one final wash with 1ml cold buffer MB without Protease and Phosphatase inhibitors. For each washing step the beads were incubated on a rotating wheel for 10min. LexA-Chromatin ring complexes were released by proteolytic cleavage of the LexA-TAP fusion protein by overnight incubation with 2 μl (purification of a single yeast strain) or 15 μl (purification of all yeast strains together) 6xHis-tagged recombinant TEV protease (NEB) in a total volume of 300 μl (purification of a single chromatin domain) or 400 μl (purification of pooled chromatin domains) in MB without Protease and Phosphatase inhibitors. Finally, beads were separated from the eluate containing the chromatin rings. DNA and protein samples were taken from beads (B) and final elution (E) (0.1% and 0.05%, respectively).

### Specific chromatin domain isolation with affinity purification: *DNA analysis*

DNA samples from the purification process were supplemented with H_2_O to a final volume of 100 μl. Additionally, 1.13 ng of a spike-in plasmid DNA (K71) were added in each sample as control for the DNA extraction efficiency. 100 μl of IRN buffer (50 mM Tris–HCl pH 8, 20 mM EDTA, 500 mM NaCl) and 2 μl RNAse A (10mg/ml) were added to the DNA samples. After incubation at 37°C for 1 h, 5 μl Proteinase K (10 mg/ml) and 10 μl of SDS 20% were added and incubated for an additional 1 h at 56°C. Subsequently, 200 μl phenol:chloroform:isoamyl alcohol (25:24:1, v/v) was added, followed by 2 × 10 s thorough vortexing. The organic and aqueous phases were separated by centrifugation for 7 min at 21 000 × g. The supernatant was transferred to a fresh 1.5 ml tube containing ethanol (2.5 : 1) and 2 μl glycogen (10mg/ml). The tube was left at −20°C overnight. The solution was centrifuged with 21 000 × g at 4°C for 45 min. The supernatant was discarded and 150 μl of 70% ethanol was added to the pellet. After another centrifugation step with 16 000 × g at 4°C for 10 min, the resulting DNA pellet was dried at room temperature for 10 min. DNA pellets were resuspended in H_2_O and digested with BsrGI restriction endonuclease (NEB) overnight at 37°C in a total volume of 35 μl. DNA content was then analyzed by qPCR or Southern blot analysis.

### Specific chromatin domain isolation with affinity purification: *protein analysis*

Protein samples were resuspended in 50 μl 1× SDS sample buffer and boiled at 95°C for 3 min. Whole cell extracts were separated by SDS-PAGE, transferred onto polyvinylidene difluoride membranes (Immobilon®-P PVDF Membrane, Sigma-Aldrich) and blocked in 5% skimmed milk dissolved in 0.05% Tween/PBS (PBST) for 1 h at room temperature. Membranes were incubated with primary antibody Peroxidase Anti-Peroxidase Soluble Complex antibody produced in rabbit for detection of TAP-tagged proteins (Sigma-Aldrich, P1291-500UL) 1:1000 dilution in 5% skimmed milk/PBST overnight at 4°C followed by washing in 0.1%Tween/TBS. Membranes were developed by chemiluminescence.

### Psoralen crosslinking

The 400 μl eluate containing the purified chromatin rings was transferred into two wells of a 24-well culture plate. Each sample was supplemented with 10 μl Trimethylpsoralen (TMP) (0.2 mg/ml in Ethanol), mixed and incubated in dark on ice for 5 min. Samples were positioned 5 cm away from 366-nm ultraviolet bulbs in a Stratalinker and then irradiated for 5min. Additional irradiations for 6 min, 7 min, 8 min were performed and before each irradiation, additional 10 μl TMP were added. After irradiation, the samples were transferred to microtubes, respective wells washed once with 200 μl IRN and the two samples combined (total volume 400μl). After treatment with RNaseA (at a final concentration of 0.33 mg/ml for 2 h at 37°C), Proteinase K and SDS were added to a final concentration of 0.33 mg/ml and 0.5% and incubation was continued for 4 h at 55°C. DNA was extracted with phenol/chloroform and precipitated overnight at 4°C. The DNA was then digested with 3 μl NcoI restriction enzyme (NEB) in 23 μl total reaction volume.

### Denaturing spreading and analysis by electron microscopy (EM)

The procedure was performed as recently described (39). For denaturing spreading, the reaction consisting of 2.0 μl formamide, 0.4 μl glyoxal and 2 μl of chromatin rARS locus was incubated for 10 min at 42°C in a thermo-mixer and subsequently transferred to an ice water bath. After this denaturation step, the reaction was spread onto carbon-coated 400-mesh magnetic nickel grids over a water surface using the benzyldimethylalkylammonium chloride (BAC) method. Following this spreading procedure, the DNA was platinum coated by platinum-carbon rotary shadowing (High Vacuum Evaporator MED 020; Bal-Tec) to render it electron dense. The grids were scanned using a transmission electron microscope (Tecnai G2 Spirit; FEI; LaB6 filament; high tension ≤ 120 kV) and pictures were acquired with a side mount charge-coupled device camera (2600 × 4000 pixels; Orius 1000; Gatan, Inc.). The images were processed with DigitalMicrograph Version 1.83.842 (Gatan, Inc.) and analyzed using ImageJ64.

### Chromatin and DNA methylation

The final elution from pooled chromatin purifications (∼200 ng total DNA in 400 μl) was supplemented with 100ng of *in vitro* assembled nucleosomal array and 1 μg of naked plasmid DNA (K112), which were used as control for methylation efficiency and nucleosome detection. Chromatin and naked DNA were treated first with 200 U of EcoGII and 200 U of M.CviPi (NEB) in the presence of S-adenosylmethionine (SAM) (NEB) at 0.6 mM and sucrose at 300 mM, and then incubated at 30°C for 7.5 min. After this incubation, additional 100 U of each enzyme and 0.05 mM SAM were added and the incubation was continued for 7.5 min at 30°C. Subsequently, 120 U of M.SssI (NEB), 10 mM MgCl_2_ and 0.05 mM SAM were added to the reaction with continued incubation at 30°C for 7.5 min. Additional 0.05 mM SAM were added and incubation at 30°C continued for 7.5 min. Methylation reaction was stopped by Stop Buffer (20 mM Tris–HCl pH 8.5, 600 mM NaCl, 1% SDS, 10 mM EDTA) in a 1:1 ratio, followed by DNA extraction as described below. All reactions were performed in low-binding reaction tubes.

### Chromatin and DNA methylation: *DNA extraction*

The methylated fragments were incubated with 2 μl RNAse A (10mg/ml) for 1 h at 37°C and 5 μl Proteinase K (10mg/ml) followed by 1 h incubation at 56°C. Subsequently, 200 μl of Phenol:Chloroform:Isoamyl Alcohol (25:24:1, v/v) was added, followed by 2 × 10 s thorough vortexing. The organic and aqueous phases were separated by centrifugation for 7 min at 21 000 × g. The supernatant was transferred to a fresh 1.5 ml tube containing ethanol (2.5:1) and 2 μl glycogen (10mg/ml). The tube was left at −20°C overnight. The solution was centrifuged with 16 000 × g at 4°C for 45 min. The supernatant was discarded and 150 μl of 70% ethanol was added to the pellet. After another centrifugation step with 16 000 × g at 4°C for 10 min, the supernatant was again discarded. The DNA pellet was dried at room temperature for 10 min. The dried pellet is then resuspended in 40 μl H_2_O and digested with 2 μl BsrGI (NEB) in a total volume of 50 μl for 2 h at 37°C. After digestion, DNA was cleaned using PCR purification kit (ThermoFisher Scientific). In the final step, DNA was eluted into nuclease-free H_2_O in a total volume of 50 μl and used for library preparation.

### Library preparation

For MATAC-Seq, the recovered DNA was converted into libraries using Ligation Sequencing Kit 1D (Oxford Nanopore Technologies, SQK-LSK109) following the manufacturer's instructions. Nanopore sequencing was carried out on R9.4 MinION flowcells (Oxford Nanopore Technologies) for up to 24 h.

### Nanopore basecalling

Megalodon (including megalodon 2.4.0, guppy 5.0.11, minimap 2.17 and res_dna_r941_min_modbases-all-context_v001.cfg) was used for base calling and detection of CpG and m6A methylation. Thresholds for binarizing the methylation states were set by generating density plots of methylation scores of 10 million methylation sites analyzed on unmethylated and fully methylated plasmid DNA samples, respectively. The cutoff score was determined separately for m6a and CpG methylation at a stringent cutoff that will result in a low false-positive calling of methylated bases yielding high specificity (Figure S5A, B).

### Bioinformatic analysis of nanopore sequencing data: *normalization*

Binary count matrices were normalized to the average methylation levels of the spiked-in *in vitro* assembled nucleosomal arrays to account for sample-specific differences in methylation efficiency. We then pooled replicates from the same condition and calculated mean methylation levels per nucleotide position (Figure 7A–D).

### Selecting significantly different chromatin regions between wildtype and mutants

We divided each origin in windows of 30 nucleotides and tested for differences in methylation levels between wildtype and CRE mutants with the paired Wilcoxon-rank-sum test. Windows with significant differences (*P*-value < 0.025) in one of the mutant strains are highlighted in gray in Figure 7A–D. We then selected these windows for comparing average methylation levels in Fig. S8E-H and for subsequent clustering analysis.

### Clustering analysis

We summarize methylation levels per 30bp window (Figure 7A–D) with the mean of normalized methylation levels for each window and each molecule. To find archetypes of methylation patterns, we first scaled the summarized methylation levels of reads from the positive DNA strand to zero mean and a standard deviation of one. We calculated the matrix of distances between molecules as $\sqrt {( {1 - \rho } )/2},$ where ρ is the Spearman's correlation coefficient of the methylation levels of windows between pairs of molecules. We applied hierarchical clustering to the distance matrix using R’s hclust and the ward. D2 method with default parameters. We applied the Elbow method (40) by calculating the Within-Cluster-Sum of Square (WSS) for different number of clusters (*k*) and selecting the k for which change in WSS first starts to diminish. Based on this analysis, we cut the dendrogram into five clusters using the function cutree. The heatmaps in Figure 8 were produced using the R package ComplexHeatmap and show average normalized methylation per window and molecule, as well as the assigned cluster number. We show the experimental condition of molecules (wildtype control, ies6Δ, isw2Δ) as a row color code. Moreover, we display the proportion of molecules per condition and cluster in the histograms in Figure 8E–H.

## Results

### ISW2 and INO80 chromatin remodelers affect replication efficiency of selected EE and LI replication origins

To test the hypothesis that nucleosome occupancy or positioning affects replication efficiency of individual origins, we first constructed isogenic CRE mutant cells deleted for subunits of the ISW2 (isw2Δ) and INO80 (ies6Δ) remodeling complexes. Cell viability assays in the presence of 100 mM hydroxyurea (HU) and 0.05% Methyl methanesulfonate (MMS) confirmed the selective sensitivity of the ies6Δ mutant strain (Fig. S1A), consistent with previous literature (41,42). We focused on four selected replication origins on yeast chromosome III, which display previously characterized major differences in their replication timing and efficiency properties (43,44) (Figure 1A). We analyzed their replication timing properties in wildtype and CRE mutant strains using a quantitative PCR (qPCR)-based DNA copy number assay (Figure 1B, and (45)). Briefly, cells are arrested in G1-phase using α-factor and then synchronously released into S-phase. In this analysis, the early-efficient (EE) ARS305 and ARS315 and late-inefficient (LI) ARS313 and ARS316 origins over a late replicating region on chromosome IV (46) showed the expected early or late replication profiles in wildtype cells (Figure 1C–F). It is important to note that such DNA copy number-based assays are bulk population measurements of the percentage of duplicated versus non-duplicated DNA of a given locus. Therefore, changes in both replication timing and efficiency of the underlying origins can affect the measured replication profiles. For this study, we will refer to higher or lower replication efficiency to describe observed relative changes in the DNA copy number profiles. Interestingly, both CRE mutant strains displayed lower replication efficiency at both EE origins. For example, ARS305 copy number dropped by 19% in isw2Δ and 18% in ies6Δ 32 min after release (Figure 1C). Similarly, ARS315 copy number dropped by 23% in isw2Δ and 18% in ies6Δ 40 min after release (Figure 1D). Interestingly, the LI origin ARS313 displayed 20% higher replication efficiency in ies6Δ at 32 min, whereas a smaller, not significant increase was observed for the isw2Δ mutant (Figure 1E). In contrast, the replication profile of ARS316 was unaffected in both CRE mutant strains (Figure 1F). Importantly, growth curves and FACS profiles showed that these changes in replication efficiency were not caused by growth defects in full medium (Supplementary Figure S1B) or differences in global arrest and release kinetics between wildtype and mutant strains (Supplementary Figure S1C, D). In addition, the observed changes are not due to an advance of replication timing of the chromosome IV control region as this region maintained a constant DNA copy number ratio of 1 in both WT and CRE mutant strains until the last 40 and 48min timepoints of the timecourse (Supplementary Figure S1E). In addition, we obtained similar results from two other unrelated late-replicating regions on Chr. V:536kb and Chr. XIV:222kb (46) (Supplementary Figure S1E). Together, these results suggested that ISW2 and INO80 CREs do not globally change the replication program and S phase progression but impact the replication efficiency of individual origins.

### Bulk chromatin accessibility analysis shows large differences between origins and ies6Δ mutant cells

**Figure 2.**
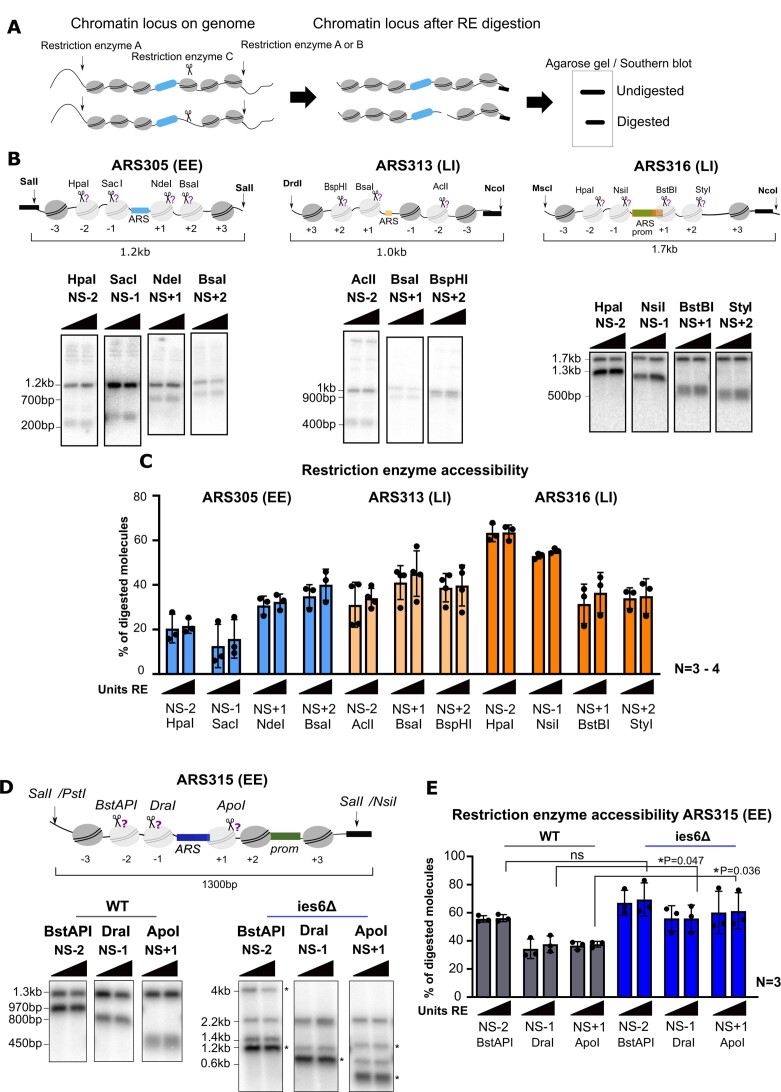
(**A**) Cartoon showing the principal of Restriction Enzyme Accessibility (REA) assay. (**B**) Restriction endonuclease accessibilities in chromosomal ARS305, ARS313 and ARS316 locus. Nuclei from yeast strains Y65 (ARS305), Y94 (ARS313) and Y69 (ARS316) were isolated and digested with increasing amounts (10U, 50U or 100U) of the indicated restriction enzymes (triangle on top of each pair of panels/scissors). DNA was isolated, digested with SalI in ARS305, KpnI in ARS313 and MscI/NcoI in ARS316 and subjected to indirect end-labeling Southern blot analysis with appropriate radioactively labeled probes. (**C**) The histogram shows the results of Southern blot quantification as a percentage of digested chromatin locus. Average and standard deviations are from *n* = 3 or 4 biological replicates (**D**) REAs in chromosomal ARS315 locus. Nuclei from yeast strains Y91 (control-ARS315), and Y130 (ies6Δ-ARS315) were isolated and digested with increasing amounts (10, 50 and 100 U) of the indicated restriction enzymes (scissors). DNA was isolated, digested with SalI and subjected to indirect end-labeling Southern blot analysis with the appropriate radioactively labeled probe. Top: schematic representation of the ARS315 locus with restriction sites used to probe chromatin structure (scissors) and secondary restriction sites to isolate the locus. Asterisks in the mutant strain indicate restriction bands derived from a spike-in naked plasmid control to verify complete digestion of the enzymes (see also Supplementary Figure S2). (**E**) The histogram shows the results of Southern blot quantification as a percentage of digested chromatin locus. Average and standard deviations are from *n* = 3 biological replicates (*, **, *** indicates statistical significance *P* < 0.05, or *P* < 0.01, or *P* < 0.001, respectively, unpaired t-test).

As a first approach to measure bulk nucleosome occupancy at our selected replication origins, we applied classical restriction enzyme (RE) accessibility assays (47–49) (Figure 2A) at individual positions expected to be protected by NS-2, NS-1, NS + 1 or NS + 2 nucleosomes around the ARS (Figure 2B). For this study, we define the +1 nucleosome of an origin as the nucleosome that is closest to the annotated T-rich strand of the ACS where the initial binding of ORC occurs. Nuclei from logarithmically growing wildtype cells were digested at different RE concentrations to establish saturation and analyzed by Southern blot analysis with specific probes directed against the selected EE and LI origins. In the wildtype strain, we observed similar percentages of digested molecules (∼30–40%) at the NS + 1 and NS + 2 restriction sites among all 4 origins (Figure 2C–E), indicating that around 30–40% of all molecules were not protected by nucleosomes at these two positions. Interestingly, we observed major differences in RE accessibility between origins at the NS-2 and NS-1 positions. For example, the NS-2 HpaI site only showed 20% digestion at ARS305, whereas more than 60% of molecules were digested by HpaI in the same relative NS-2 position of ARS316 (Figure 2C). We also note that the ARS305 NS-1 position showed strong protection (∼10% digestion), whereas more than 50% of the molecules were accessible at this site in ARS316 (Figure 2C), consistent with a strongly positioned NS-1 nucleosome at the EE origin ARS305 (21). It is important to note that these RE accessibility differences are observed at the four origins in the context of the wildtype strain and are not related to accessibility changes of the same origin between wildtype and CRE mutant strains as analyzed below.

To this end, we asked whether knockout of IES6 led to changes in absolute nucleosome occupancy at selected sites around ARS315 (Figure 2D, E). In the ies6Δ samples, we also included in the RE accessibility assay a naked spike-in plasmid DNA containing the same ARS315 origin DNA sequence and observed 100% digestion of the naked DNA into the expected DNA fragments (Figure 2D, DNA bands marked with asterisks and Supplementary Figure S2), confirming the reactivity of the used REs. Thus, it can be concluded that the fraction of remaining undigested molecules from the nuclei was caused by the protective chromatin structure of the ARS315 locus. We observed no significant difference in accessibility between wildtype and ies6Δ strain at the NS-2 BstAPI restriction site located 222 bp away from the ARS (Figure 2E). However, the ApoI and DraI sites flanking the ARS in NS + 1 and NS-1 positions showed an 18% and 24% increase in accessibility, respectively (Figure 2E), suggesting that upon loss of INO80, the typically well positioned nucleosomes in close vicinity to the ARS are evicted or repositioned, thereby making this site more accessible in the mutant strain. Together, these bulk analyses suggested an inherent level of heterogeneity in nucleosomal occupancy at individual positions and that INO80 can affect chromatin accessibility at ARS315.

### Functional replication origins are purified with high yield and purity in their native chromatin context

To obtain a more comprehensive readout of chromatin accessibility and nucleosome occupancy changes, we sought to investigate potential local and global changes of the chromatin landscape of the targeted replication origins between CRE mutant and wildtype strains. Ideally, the method of choice should allow for quantitative profiling of the accessibility of individual chromatin fibers at the single-molecule level with high resolution and coverage, thereby revealing the heterogeneity of multiple chromatin states that may co-exist in a cell population. For this, we developed Methylation Accessibility of TArgeted Chromatin domain Sequencing (MATAC-Seq), a single-molecule approach to profile the chromatin structure of a targeted locus of interest at near basepair resolution. MATAC-Seq is built on the conceptual foundations of NOMe-Seq and SMAC-Seq (50–54) that rely on the preferential modification of accessible DNA by DNA methyltransferases combined with Nanopore sequencing for direct readout of the methylated DNA bases. In MATAC-Seq, we additionally take advantage of our previously established single-copy locus chromatin purification approach (38,45,55,56). This strategy allows strong enrichment for our targeted replication origins prior to methylation footprinting and Nanopore sequencing analysis, thereby overcoming current limitations of DNA sequencing coverage of similar genome-wide approaches (53).

**Figure 3.**
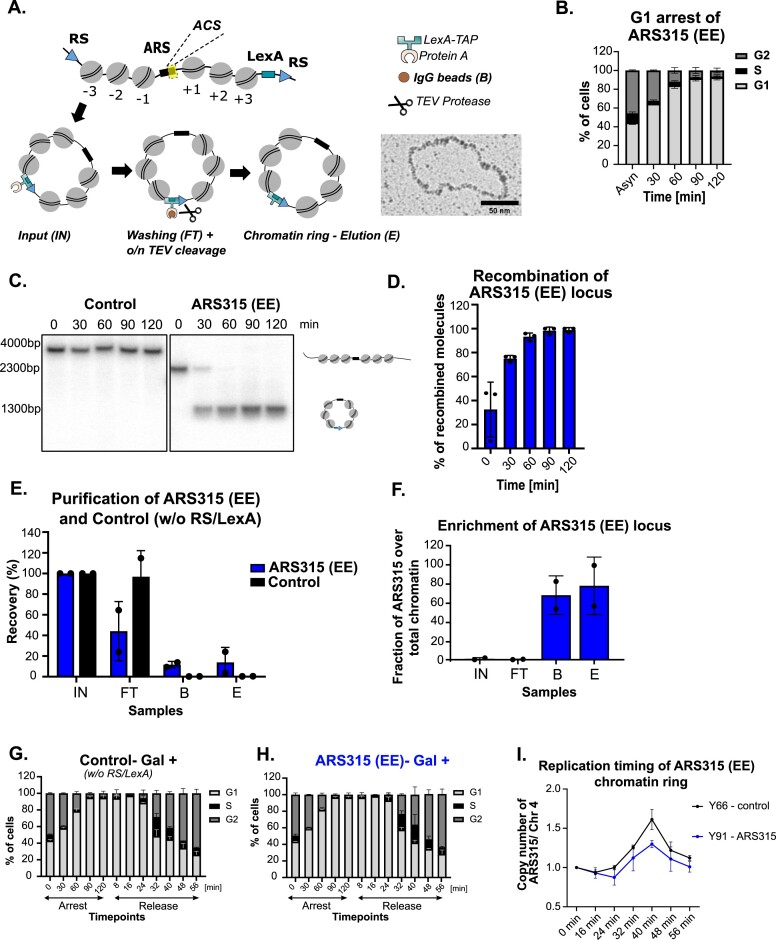
(**A**) Schematic representation of LexA affinity purification. Recombination sites (RS) were integrated after the ±3 nucleosomes around the ARS locus of single copy origins and after the ±2 nucleosomes of the multi-copy rARS origin. Electron micrograph shows individual ribosomal ARS molecule after native spreading. Scale bar = 50 nm. (**B**) Cell arrest in G1-phase. The strain Y91-ARS315 was grown in YPR medium and arrested with 50 ng/ml α-factor. Samples for FACS analysis were taken at the indicated timepoints and stained with Sytox green to monitor the distribution of G1, S and G2-phase in both profiles. (**C**) Recombination kinetics of ARS315 locus. The strains Y91-ARS315 and Y66-control (w/o RS or LexA sites) were grown in YPR medium to logarithmic phase and arrested in G1-phase by addition of α-factor (50 ng/ml) and recombination was induced by the addition of 2% galactose. Samples were taken at indicated timepoints. DNA was isolated and linearized by BsrGI and subjected to Southern blot analysis. The positions of unrecombined and recombined molecules are shown on the right. (**D**) The histogram shows the results of Southern blot as percentage of recombined chromatin locus. (**E**) LexA affinity purification was performed for strain Y91, where the single-copy ARS315 locus is targeted by RS, and strain Y66 (control), lacking RS/LexA recognition sites but containing the LexA expression cassette, therefore no recombination or purified chromatin is expected. DNA samples were taken (0.1% Input (IN), Flowthrough (FT), Beads (**B**), Eluate (**E**) from *n* = 2 biological replicates and analyzed by qPCR. The enrichment of an unrelated region (PDC1) was analyzed side-by-side with the region of interest. (**F**) Enrichment of ARS315 locus in the final eluate. Given that the size of yeast genome is 12 kb, and the length of a chromatin ring is ∼1 kb, the fold enrichment ratio of the specific origin to PDC1 was used to define the enrichment of an origin in the DNA samples. (**G–H**) Cell cycle progression of control (Y66) (w/o RS/LexA sites) and ARS315 (Y91-with RS/LexA sites) strains after addition of 2% galactose. The cells were arrested by 50 ng/ml α-factor and released into S phase by addition of 125U Pronase. (**I**) The replication timing plots show the average copy number ratios after addition of 2% galactose of ARS315 locus, either in the Control (Y66) strain containing no RS/LexA sites for recombination or in the ARS315 (Y91) containing RS/LexA sites, to the late replicating region of ChrIV with standard deviation from *n* = 2 biological replicates. The cells have been arrested for 2 h by 50 ng/ml α-factor and released to S-phase by addition of 125 U Pronase.

Briefly, site-specific recombination allows excision of native chromatin rings encompassing a ∼1 kb genomic region centered around the single-copy replication origins from its endogenous chromosomal location in G1-phase arrested cells (Figure 3A-B). The necessary insertion of RS/LEXA sites had no impact on growth and generation timing in all recombination-competent strains compared to the control strain (Supplementary Figure S3A). We monitored the recombination kinetics and efficiency under these conditions in a time-course experiment by Southern blotting of extracted genomic DNA in a negative Control strain (no RS/LEXA sites) and the recombination-competent ARS315 and ARS313 strains. We observed near complete recombination of the targeted locus within ∼60–90 min after recombinase induction (Figure 3C, D and Supplementary Figure S3B–D). Cells were lysed and the whole cell extract subjected to a single-step affinity purification protocol using the LexA-TAP protein as an affinity handle (Figure 3A). We took DNA samples from the resulting Input (IN), Flowthrough (FT), Beads (B) and Elution (E) fractions and quantified the amount of ARS315 DNA by qPCR (Figure 3E). After Tobacco Etch Virus (TEV) protease-mediated cleavage of LexA-TAP on the beads, ∼15% of all chromatin rings relative to the input were recovered in the final elution sample, whereas no enrichment of ARS315 was observed in the control strain. Indeed, the purified eluate fraction showed a strong enrichment of ARS315 over an unrelated control locus (PDC1) and we estimate that ∼70–80% of all DNA molecules in the final eluate were derived from our targeted locus (Figure 3F).

Importantly, we verified that our genetic manipulations and the necessary process of recombination did not interfere with functionality of the origin. To this end, control cells without RS/LEXA and ARS315 cells were arrested in G1-phase using α-factor with addition of Galactose (Gal +) to induce recombination in the ARS315 strain and then released synchronously into S-phase. FACS analysis showed highly similar arrest and release kinetics between the two strains (Figure 3G, H). We then performed DNA copy number analysis at different timepoints after release by qPCR comparing the replication timing of ARS315 in its genomic location (Control, black) versus in its excised state as a chromatin circle (ARS315, blue) (Figure 3I). Importantly, when comparing the ratio of ARS315 replication with a late replicating region on chromosome IV (Chr4, (46), see also Figure 1B), the two replication profiles showed similar kinetics where ARS315 started replicating between 24 to 32 min and DNA copy numbers increased. After ∼40 min release, the region on ChrIV also started to replicate and DNA copy numbers decreased in both strains, consistent with earlier replication of ARS315 compared to ChrIV. We note that the excised ARS315 chromatin circles did not reach the same maximum DNA copy number ratio at 40min as the Control genomic ARS315, suggesting that a fraction of chromatin circles did not replicate as efficiently as in its genomic context which could stem from the lack of stochastic firing of neighboring origins and passive replication when isolated from its genomic context.

We also tested whether the process of recombination and purification affected chromatin accessibility at individual sites on the chromatin circles by restriction enzyme accessibility assays. Importantly, purified ARS305 and ARS313 chromatin rings (eluate) and isolated nuclei from the same strains before and after recombination displayed similar sensitivities to restriction endonucleases, suggesting that the chromatin structure was not affected (Supplementary Figure S3E, F), as previously demonstrated for other chromatin domains purified by this approach (38). We additionally asked whether the simultaneous excision of chromatin rings during the α-factor mediated G1-arrest has any impact on the chromatin state of the rings as in principle, the chromatin domains are excised during different cell cycle stages by this approach. To address this, a side-by-side comparison between simultaneous excision and G1-arrest versus sequential G1-arrest followed by chromatin ring excision of an early (ARS315) and a late (ARS316) origin domain was performed (Supplementary Figure S4A). Comparing the restriction enzyme accessibility of several sites on the chromatin under both conditions, our results consistently demonstrated that the timing of chromatin ring excision versus G1-arrest did not exhibit significant changes in chromatin accessibility on the chromatin domains (Supplementary Figure S4B, C). Together, we conclude that excision of the targeted origin from its genomic context does not interfere with the major chromatin properties of the origins and therefore also preserves the intrinsic replication state of the origin.

### MATAC-Seq and Psoralen-crosslinking electron microscopy show comparable single-molecule nucleosomal profiles on the multicopy ribosomal ARS domain

**Figure 4.**
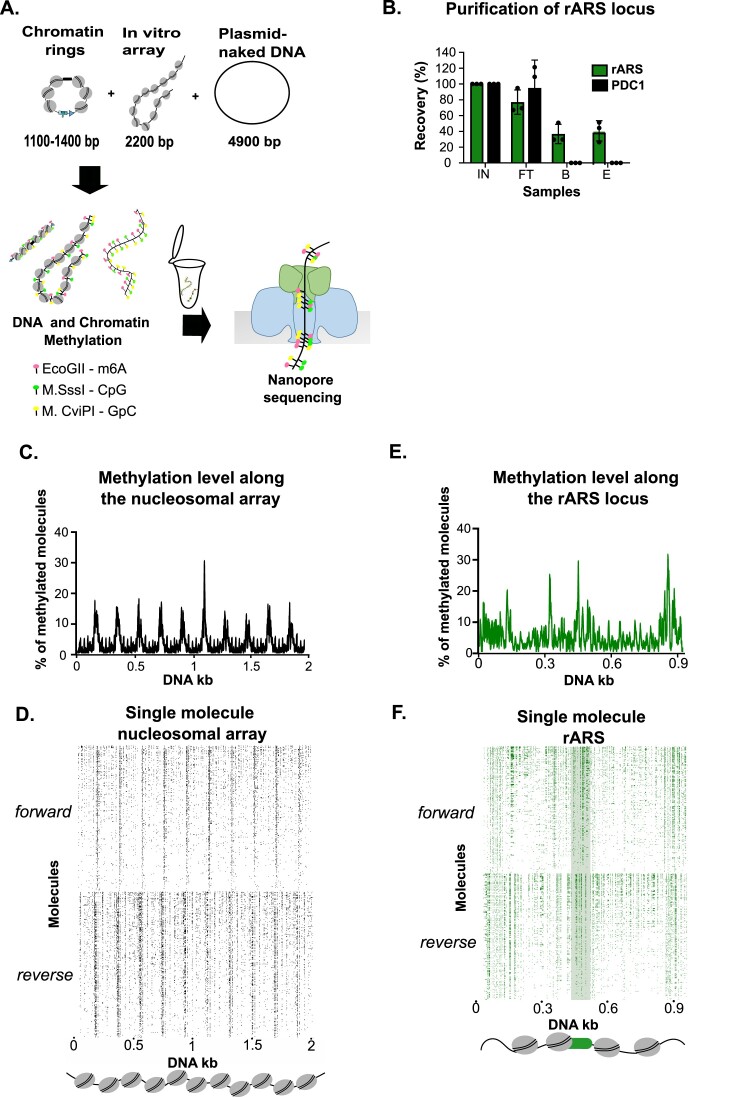
(**A**) Experimental outline of single molecule Methylation Accessibility TArgeted Chromatin loci sequencing assay (MATAC-seq). The purified chromatin rings and two spike-in controls; an *in vitro* assembled nucleosomal array and a naked plasmid were treated with m6A, CpG and GpC methyltransferases, which preferentially methylate only accessible DNA bases. DNA was isolated and the circular molecules were linearized with BsrGI and subjected to Nanopore sequencing. (**B**) LexA affinity purification was performed for strain Y84, where the multi-copy rARS locus is targeted by RS. DNA samples were taken (0.1% Input (IN), Flowthrough (FT), Beads (B), Eluate (E) from *n* = 3 biological replicates and analyzed by qPCR. The enrichment of an unrelated region (PDC1) was analyzed side-by-side with the regions of interest. (**C**) Average methylation profile of *in vitro* assembled nucleosomal array. MATAC-seq reveals the repetitive pattern of protected nucleosomal regions separated by narrow hyper-methylated linker sites. (**D**) MATAC-Seq reads of the *in vitro* assembled nucleosomal array. Each row represents a single molecule displaying methylation events at near bp resolution. The reads have been ranked according to their methylation level (higher on the top and lower on the bottom) on both forward and reverse DNA strands. (**E**) Average methylation profile on the native rARS locus reveals high accessibility at the centrally positioned ARS region. (**F**) MATAC-Seq reads of the multi-copy rARS chromatin locus. Each row represents a single molecule displaying methylation events at near bp resolution. The reads have been ranked according to their methylation level (higher on the top and lower on the bottom) on both forward and reverse DNA strands. Identical number of reads were used per DNA strand and per sample (1560 reads) in MATAC-seq plots.

We next used a combination of M.CviPI/M.SssI (GpC/CpG-5-methyl-Cytosine-specific) and EcoGII (methyl-6-Adenine-specific) DNA methyltransferases, which catalyze preferential methylation of accessible DNA bases. Direct detection of methylated nucleotides by Nanopore sequencing allowed us to generate single-molecule readouts of the chromatin accessibility states of individual ARS domains. In addition, we spiked-in an *in vitro* reconstituted nucleosomal array with 12 × 601 nucleosome positioning sequences (57) and a naked plasmid DNA control into the reaction to determine overall DNA methylation efficiencies and variability between individual biological replicates (Figure 4A). To establish the assay and optimize the methylation conditions, we first focused on the multi-copy ribosomal ARS locus, which could be purified in large quantities and high yields using the same site-specific recombination and affinity purification approach as previously shown (Figure 4B and (38)). The nucleosomal array revealed a methylation pattern that was consistent with highly positioned nucleosomes formed over the 601 sequences and intervening accessible linker DNA (Figure 4C, D). In contrast, the methylation pattern of the purified rARS domain, as an example of a native chromatin domain, was much more irregular without equally spaced peaks (Figure 4E, F). At the center of the individual chromatin rings, a highly methylated and thus accessible region was clearly visible that coincided with the annotated position of the rARS NFR. In addition, the flanking regions around the rARS displayed higher resistance against methylation, indicating nucleosomal protection. Importantly, a regular pattern of unmethylated DNA interspersed with highly accessible linker DNA was not as pronounced as for the nucleosomal array, suggesting a much higher level of heterogeneity at this locus (Figure 4E, F).

We benchmarked this single-molecule methylation dataset with psoralen-crosslinking electron microscopy (EM), considered as a gold standard approach to analyze single-molecule nucleosome configurations (54,55,58). To this end, we purified rARS chromatin rings under identical conditions, incubated the native chromatin with trimethylpsoralen and exposed to UV-A light, which is known to result in interstrand-crosslinked DNA in linker but not nucleosomal DNA. Thus, positions previously occupied by nucleosomes are visible in denaturing electron microscopy as single-stranded DNA bubbles, separated by double-stranded linker DNA that resist denaturation due to psoralen crosslinking (Supplementary Figure S5A). DNA was linearized such that the nucleosome-free ARS region was in the center of the molecule and the positions of the observed surrounding DNA bubbles were mapped in all molecules (Supplementary Figure S5B). Overall, we detected 143 molecules that strictly conferred to the expected size range of ∼1kb (Supplementary Figure S5C). Importantly, the majority of detected bubbles (∼70%) on the rARS molecules displayed the expected size range of mono-nucleosomal bubbles (Supplementary Figure S5D, light blue area). Of note, the residual 30% of the bubbles showed smaller (<135 bp), intermediate (165–300 bp) or di-nucleosomal sizes, presumably because of incomplete crosslinking of naked DNA due to sequence preferences for psoralen-crosslinking, protection by chromatin components other than nucleosomes or inefficient linker DNA crosslinking between two nucleosomes (Supplementary Figure S5D). Interestingly, the molecules revealed substantial heterogeneity in the number, position, and size of DNA bubbles, consistent with our methylation footprinting analysis (Supplementary Figure S5E). These data suggest the co-existence of a variety of chromatin states at this locus. Unlike MATAC-Seq, the EM dataset did not allow to orient individual molecules. Thus, we could only directly compare the accessibilities between the two methods at the two ends and the centrally positioned ARS of the linearized molecules. Importantly, overlapping the bulk profiles generated from psoralen-EM with the methylation footprinting data revealed highly concordant profiles between the high accessibility regions (high methylation and low frequency of nucleosomal bubbles), whereas the intervening regions showed higher nucleosomal protection (Supplementary Figure S5F). We conclude that MATAC-Seq is robust and provides high-resolution chromatin accessibility maps of specific chromatin domains.

### MATAC-Seq reveals major changes in origin chromatin accessibility between wildtype and CRE mutant strains

Having established a method to determine chromatin states with single-molecule resolution, we next wanted to obtain insights into the potential single-molecule differences of origin chromatin accessibility between the wildtype and CRE mutant strains. To this end, we used site-specific recombination to excise native chromatin rings encompassing a ∼1 kb genomic region centered around the single-copy ARS305, ARS313, ARS315 and ARS316 replication origins in the wildtype, isw2Δ and ies6Δ mutant strains. We first validated that the targeted purification of the 4 single-copy replication origins via LexA-TAP showed comparable purification efficiencies and enrichments between the individual strains and biological replicates (Supplementary Figure S6A–D). We note that ARS316 chromatin domains in the ies6Δ mutant and ARS313 chromatin domains in both CRE mutants were less efficiently recovered in the final eluate than for the wildtype purifications (Supplementary Figure S6Β). Despite this observation, genomic mapping of MATAC-Seq reads on chromosome III confirmed a strong enrichment and sufficient coverage of reads overlapping with the targeted replication origins and low numbers of other contaminating genomic reads (Supplementary Figure S6E).

**Figure 5.**
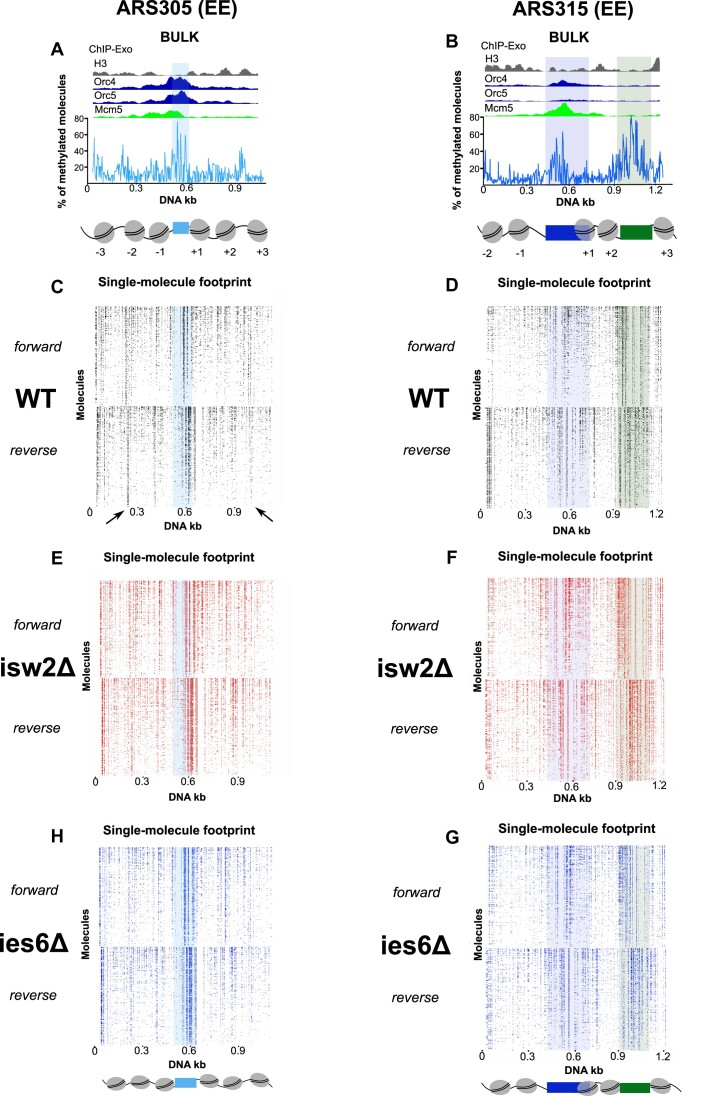
(**A**, **B**) Average methylation profile around the early and efficient (EE) replication origins (ARS305, ARS315) derived from MATAC-Seq recapitulates the known nucleosomal pattern derived from bulk ChIP-Exo (62) analysis. (**C**, **D**) Chromatin accessibility maps around the native ARS loci of wildtype strains revealing less nucleosomal ARS regions and high level of heterogeneity. The methylated DNA bases of each molecule are depicted as dots and the reads have been organized according to their methylation level (higher on the top and lower on the bottom) in both DNA strands. Identical number of reads were used for forward and reverse strands in MATAC-seq plots. (**E**, **F**) MATAC-Seq chromatin accessibility maps of the ARS regions in the isw2Δ strains. Identical number of reads were used for forward and reverse strands in MATAC-seq plots. (**G**, **H**) MATAC-Seq chromatin accessibility maps of the ARS regions in the ies6Δ strains. Identical number of reads were used for forward and reverse strands in MATAC-seq plots.

**Figure 6.**
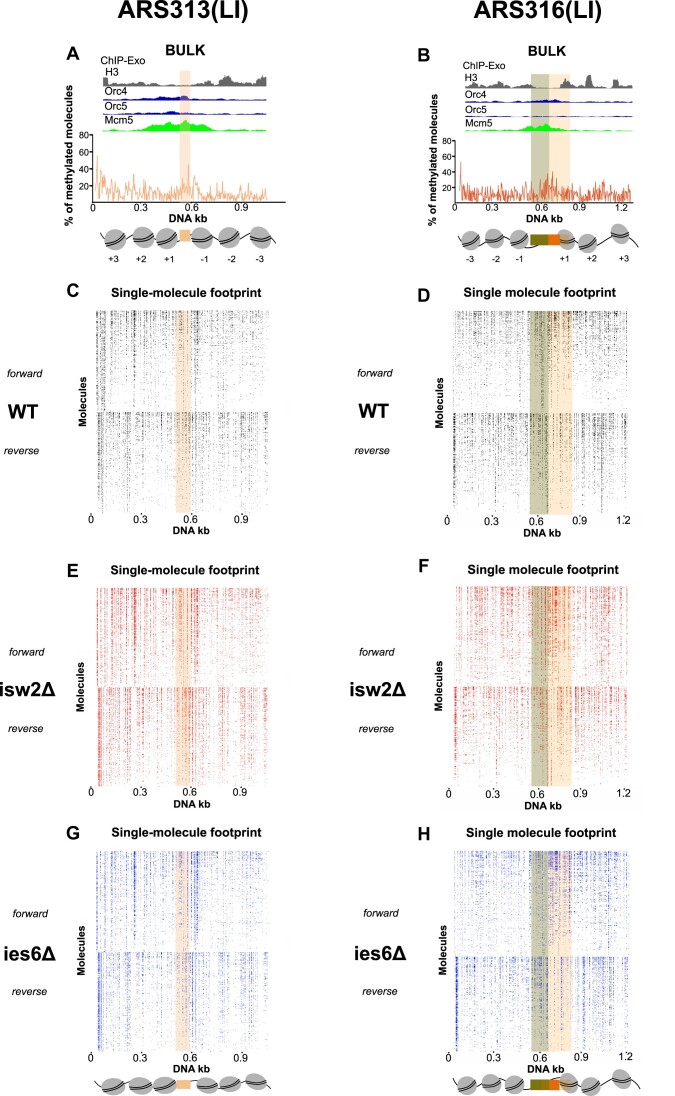
(**A**, **B**) Average methylation profile around the late and inefficient (LI) replication origins (ARS313, ARS316) derived from MATAC-Seq recapitulates the known nucleosomal pattern derived from bulk ChIP-Exo (62) analysis. (**C**, **D**) Chromatin accessibility maps around the native ARS loci of wildtype strains revealing less nucleosomal ARS regions and high level of heterogeneity. The methylated DNA bases of each molecule are depicted as dots and the reads have been organized according to their methylation level (higher on the top and lower on the bottom) in both DNA strands. Identical number of reads were used for forward and reverse strands in MATAC-seq plots. (**E**, **F**) MATAC-Seq chromatin accessibility maps of the ARS regions in the isw2Δ strains. Identical number of reads were used for forward and reverse strands in MATAC-seq plots. (**G**, **H**) MATAC-Seq chromatin accessibility maps of the ARS regions in the ies6Δ strains. Identical number of reads were used for forward and reverse strands in MATAC-seq plots.

For the single molecule analysis, we used Megalodon (59–61) to extract the CpG and m6A-methylated bases from raw Nanopore reads. We took into account an equal number of reads from forward and reverse strands and obtained a very high read depth for each origin and condition (984–1560 reads), with the exception of the ARS316 ies6Δ mutant, for which we recovered fewer reads (548 and 166 per replicate) (Supplementary Figure S6F). To reduce the noise from false positive detection of methylation, we compared the methylation density scores of an unmethylated to fully methylated naked plasmid and set highly stringent thresholds for CpG and m6A methylation (Supplementary Figure S7A, B). Importantly, we also validated the significance of our datasets by comparing the wildtype average methylation patterns generated by MATAC-Seq across all reads with bulk accessibility profiles of recently published ChIP-Exo datasets of canonical histone H3, ORC4, ORC5 and MCM5 at the two EE origins (62) (Figure 5) and the two LI origins (Figure 6). Notably, the H3-depleted ARS region of each origin was hyper-accessible and overlapped strongly with the ORC and MCM ChIP-Exo peaks (Figures 5A, B and 6A, B) and the NFR of the Tvs1 gene promoter downstream of ARS315 showed pronounced accessibility (Figure 5B), further confirming that MATAC-Seq can reliably identify open regulatory chromatin regions.

We first ranked all single-molecule forward and reverse reads according to their total methylation level in the wildtype, isw2Δ and ies6Δ mutant (Figures 5C–G and 6C–G). In the wildtype maps, multiple locations (for example at the –3 and + 3 nucleosomal regions of ARS305) displayed strongly positioned nucleosomal footprints with nearly all individual reads exhibiting an accessible linker region between the nucleosomes (Figure 5C, black arrows). However, most visible substructures exhibited considerable heterogeneity with a gradient in methylation levels along both strands, suggesting an overall diverse protein occupancy landscape likely generated by the co-existence of multiple chromatin states on individual molecules. Notably, the WT strain showed on average a 22% larger MATAC-Seq peak height of methylation at the ARS regions at the two EE origins ARS305 and ARS315 compared to the two LI ARS313 and ARS316 origins (Figures 5A, B and 6A, B, see shaded boxes at the center of the bulk MATAC-Seq profiles). The higher accessibility in the NFR regions of the EE ARS is consistent with a more structured chromatin state at the EE origins, which likely facilitates a more regular phasing of nucleosomes and other surrounding chromatin factors.

Interestingly, the overall frequency distribution of the percentage of methylated bases was shifted towards higher methylation levels in the two mutants compared to the wildtype. This effect was consistent across all four origins and more pronounced in the isw2Δ mutant (Supplementary Figure S7C–F), suggesting that deletion of ISW2 has a stronger impact on nucleosomal occupancy/chromatin accessibility than deletion of IES6. To exclude the possibility that these differences derive from technical variations—e.g. different efficiencies of the methylation reaction—we took advantage of the spike-in naked plasmid and nucleosomal array to assess the methylation levels and allow normalization between individual experiments (Supplementary Figure S7G, H, see also Materials and methods section for details). Importantly, the spike-in naked plasmid also contained the ARS305 sequence, allowing us to directly compare the methylation efficiency of the same DNA sequence in the context of naked DNA versus native chromatin. The chromatinized molecules showed clear protection from methylation except for the central ARS position that displayed similar accessibility between the naked plasmid and native chromatin context (Supplementary Figure S7I).

### An optimal size and accessibility level of the NFR combined with well-positioned +1 and +2 nucleosomes allow efficient origin replication

**Figure 7.**
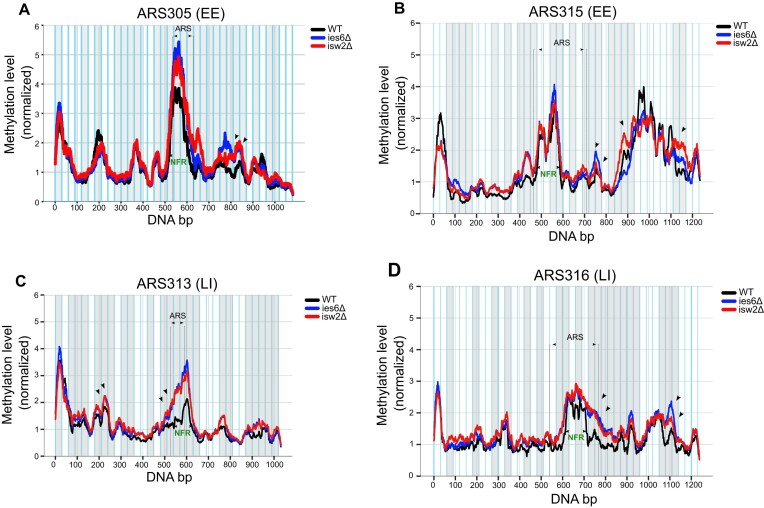
(**A**–**D**) Comparative analysis of chromatin accessibility on replication origins between WT and CRE mutant strains shows statistically significant difference on specific features. The methylation level of each condition has been normalized to the average methylation value of the respective nucleosomal array of each dataset and smoothened using 30 bp windows. Each chromatin locus has been split into 30 bp genomic bins indicated by light blue lines and the genomic bins showing statistically significant differences in DNA methylation between WT and CRE mutant strains are highlighted with light grey shading. The annotated ARS of each origin and the Nucleosome Free Regions (NFR) according to the MATAC-Seq data are depicted by dashed black lines and green arrows, respectively. The blue and red arrows highlight the chromatin regions mentioned in the text. Average and standard deviations are from *n* = 2 biological replicates.

We next asked whether the increased accessibility in the CRE mutants is due to a uniform gain of accessibility across the complete ∼1 kb regions or whether specific genetic elements within the region were more affected than others. Importantly, the methylation profiles of wildtype and CRE mutant strains were highly reproducible between the biological replicates (Supplementary Figure S7J–M), allowing us to pool the replicates and thereby increasing the statistical power. After normalization of the methylation levels of wildtype and mutant strains, we overlaid the resulting methylation profiles across the whole origin between wildtype and CRE mutant strains (Figure 7A–D, grey boxes and Supplementary Figure S8A–D).

One of the most striking changes observed consistently was that the accessibility of the ARS was significantly increased in both mutants. Importantly, this pattern was consistent for all replication origins (Figure 7A–D, ARS), suggesting that both chromatin remodelers have overlapping functions in regulating ARS accessibility, either by directly sliding nucleosomes into the ARS or indirectly by establishing a more regular chromatin structure in direct proximity of the ARS. Deletion of ISW2 increased the size of the NFR at the EE origin ARS305 and shifted the position of the linker between the +1 and +2 nucleosome further downstream (Figure 7A red arrow), consistent with repositioning of the +1 nucleosome further away from the ARS. A similar broadening of the NFR was observed for the ies6Δ mutant together with an increased accessibility between the +1 and +2 nucleosomes (Figure 7A, blue arrow). Together, these bulk methylation profile changes indicate a reduction in nucleosome array regularity at the NS + 1 and NS + 2 regions of ARS305 origin chromatin. Interestingly, the ARS315 (EE) origin showed similar changes for both mutants, with an increase in the accessibility of the linker between the +1 and +2 nucleosome compared to the wildtype (Figure 7B, red/blue arrows). In addition, the isw2Δ mutant showed a broadening of the Tvs1 promoter NFR (Fig, 7B, red arrows around Prom on the right). One interpretation of this data is that the nucleosome upstream to the Tvs1 promoter was shifted towards the ARS, thereby reducing the spacing of the +1 and +2 nucleosomes located between the promoter and ARS (Figure 7B).

ISW2 and IES6 deletion also resulted in a strong increase in accessibility at the otherwise well-protected ARS of ARS313 (LI) and ARS316 (LI), but also led to an increased size of the NFR, particularly towards the + sides of the two ARS (Figure 7C, D, red and blue arrows). Interestingly, the ARS313 NFR gained accessibility to a similar normalized methylation level (>3) as observed for the two EE origins ARS305 and ARS315 in WT cells (compare Figure 7A, B with Figure 7C). At the ARS316 origin, both isw2Δ and ies6Δ mutants broadened the NFR (Figure 7D, red and blue arrows). The enlargement of the NFR coincided with an increased accessibility downstream at two additional positions, consistent with a shift or sliding of the +1 and +2 nucleosomes in both mutants (Figure 7D).

Together, these differential accessibility patterns of the CRE mutants align with changes of the EE and LI states of the origins. First, the EE origins both decrease replication efficiency upon ISW2 or INO80 deletion (Figure 1C, D). In both cases, we observed increased accessibility at the linker position between the + 1 and + 2 nucleosomes, suggesting that the positioning or distance of these nucleosomes might have an impact on the EE replication property of these origins. Second, the LI origin ARS313 advanced replication in the CRE mutant, most prominently in the ies6Δ cells (Figure 1E). Intriguingly, the CRE mutant strains broadened the NFR and gained accessibility to similar levels as the wildtype NFR of the two EE origins ARS305 and ARS315. Finally, no advancement of replication of the LI origin ARS316 was observed in both CRE mutants (Figure 1F). Although overall accessibility levels of the ARS316 NFR was also increased for both CRE mutants compared to WT, the most dramatic change was the large broadening of the NFR into the position of the +1 nucleosome of the origin. These changes detected at the bulk level support the notion that specific chromatin features such as an optimal size and accessibility of the NFR combined with a regularly positioned +1 and +2 nucleosome can serve as a positive regulator of replication efficiency—at least at the four loci that were investigated.

### Hierarchical clustering reveals a distinct chromatin state with an open ARS and well-positioned +1 and +2 NS that correlates with efficient origin activity.

**Figure 8.**
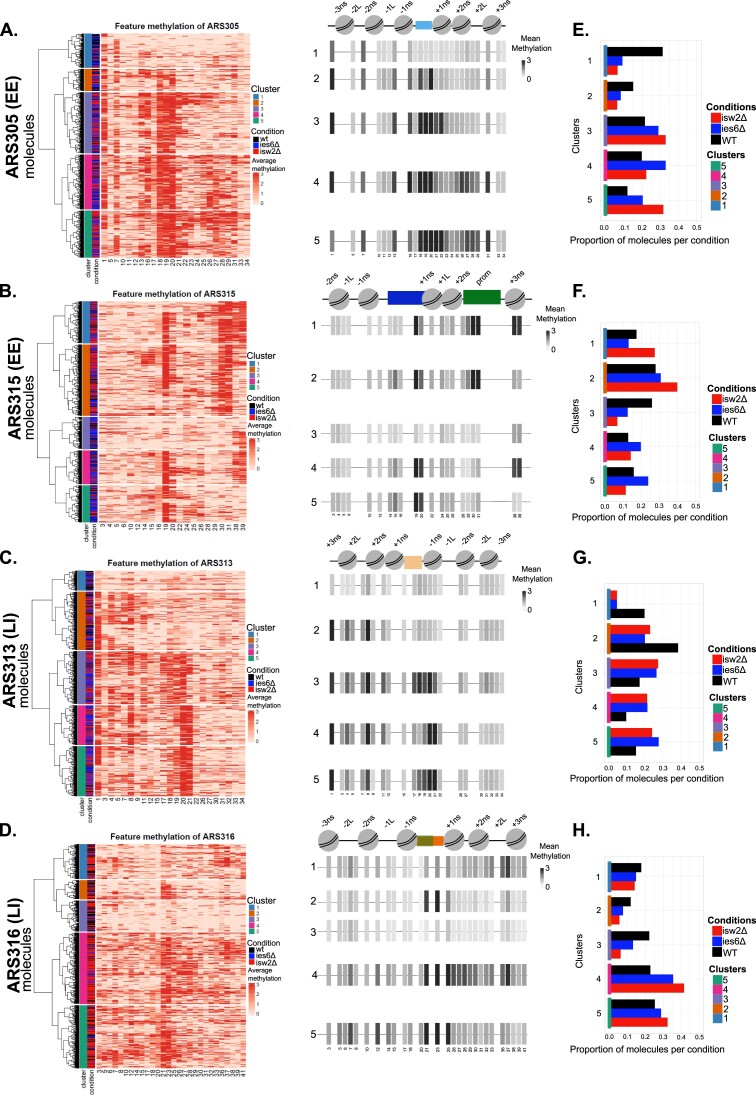
(**A**–**D**) Heatmaps showing hierarchical cluster analysis of the summarized methylation levels per feature along the single ARS molecules. For each origin, the dendrogram was cut after five clusters. The graphical representation on the right indicates only the 30 bp genomic bins showing significant difference between the WT and the CRE mutant strains. The mean methylation of each bin has been calculated and the open regions with high methylation level are depicted by grey-colored gradients. (**E**–**H**) The plots show the proportion of the reads per condition and cluster. The total number of reads has been normalized to one.

In order to leverage the power of our single-molecule datasets and identify groups of molecules with common chromatin accessibility states, we applied hierarchical clustering analysis on the molecules derived from the four replication origins in wildtype, ies6Δ and isw2Δ mutants. To this end, we divided the origins into small consecutive bins with a window size of 30bp and then determined the average methylation level for each bin and molecule (Figure 7A–D). We then determined which 30bp bins show significant changes in the methylation levels between the WT and CRE mutant samples (Figure 7A–D, grey boxes). Based on the bins with significant changes, hierarchical clustering analysis of each origin was performed. To determine the number of clusters (*k*), we applied the elbow method to the Within-Cluster-Sum of Square (WSS; see Materials and methods) and estimated that for all four origins, *k* = 5 represents the optimal choice for the number of clusters in our data (Supplementary Figure S8A–D). Therefore, we chose five distinct clusters from the hierarchical clustering analysis (Figure 8A–D), so that each cluster presented a unique chromatin accessibility state. Importantly, splitting the reads that contribute to each of the five clusters according to biological replicates showed highly similar contributions from the two independent experiments, but distinct contributions from the experimental condition per cluster (wildtype, ies6Δ and isw2Δ mutants) (Supplementary Figure S8E-I). Based on the resulting heatmaps for each origin (Figure 8A–D), we assigned for each cluster the mean methylation levels to illustrate the relative chromatin accessibility state along the origins (Figure 8A–D, heatmaps in the center). We then determined in a bar graph presentation the relative percentage of reads that contributed to each cluster from wildtype, ies6Δ and isw2Δ mutant conditions (Figure 8E–H).

MATAC-Seq revealed coordinated changes of chromatin states at different positions between wildtype and CRE mutants at the investigated EE and LI origins. Interestingly, all origins contained one cluster that was highly protected from methylation across all bins (cluster 1 in ARS305 and ARS313, cluster 3 in ARS315 and ARS316), suggesting that a fraction of the molecules exist in a fully protected nucleosomal state where even the NFR region of the ARS is protected. In ARS305 (Figure 8E), this cluster 1 is the main cluster in wildtype cells which challenges previous models that an open ARS region is a necessary and uniform requirement of an EE origin based on bulk experiments (17,21). Importantly, this chromatin state is much less frequently observed in both CRE mutant strains. A similar result was obtained for cluster 2, characterized by an open ARS surrounded by relatively well positioned nucleosomes. This chromatin state is also less frequently observed in both ies6Δ and isw2Δ mutants. In contrast, more reads contribute to clusters 3, 4 and 5 in the CRE mutants. The common feature of these three clusters is an increased accessibility of the ARS that is extended into the +1 and +2 NS. Clusters 4 and 5 show additionally high accessibility further downstream in the linker region between the +2 and +3 nucleosome, suggesting that nucleosomes are evicted or have shifted to make this region of the origin more accessible. Given that the CRE mutants show an overall decreased replication efficiency of ARS305 (Figure 1C), this cluster analysis indicates that a defined NFR at the ARS combined with a regular nucleosomal structure at the +1 and +2 NS is a chromatin state that supports or at least highly correlates with early and efficient replication of the ARS305 origin.

In ARS315 (Figure 8B and F), all clusters except for the previously mentioned cluster 3 show a defined, accessible NFR at the ARS region. Clusters 1 and 2 represent chromatin states containing both an open ARS and an extended open Promoter site that is breaching into the expected positions of the +1 and +2 NS. Cluster 1 shows additionally high accessibility at the +3 NS position, suggesting even further extension of the NFR to the other side of the promoter. Interestingly, these two states are more frequently observed in isw2Δ cells (Figure 8F), consistent with a model that the extended NFR of the gene promoter interferes with regular spacing of the +1 and +2 NS of the ARS315 origin, thereby decreasing replication efficiency similar to the effect observed in the other EE origin ARS305. The ies6Δ cells more frequently contribute to clusters 4 and 5 at this origin, which are characterized by elevated accessibility on both −1 and +1 NS sides of the origins, consistent with the results from our RE accessibility assays (Figure 2E). Similarly, these data indicate that the nucleosomes surrounding the origin are not as precisely positioned and emphasize their importance towards efficient replication initiation.

ARS313 is a LI origin that advanced replication most significantly in the ies6Δ CRE mutant (Figure 1E). In wildtype cells, clusters 1 and 2 containing a closed ARS as a common feature contribute most reads to the population (Figure 8G), suggesting that the strong ARS occupancy is a limiting feature at this origin. Intriguingly, the CRE mutant strains contributed less to the clusters 1 and 2 but shifted a significant portion of reads to clusters 3, 4 and 5 that all displayed an open ARS. Most strikingly, the largest cluster in the ies6Δ mutant was cluster 5, characterized by an open ARS and reduced accessibility in the +1 and +2 NS region. This represents a highly similar chromatin state as observed in both EE origins ARS305 and ARS315, where a defined NFR at the ARS combined with a regular nucleosomal structure at the +1 and +2 NS is a chromatin state that supports or at least highly correlates with early and efficient replication of these origins (Figure 8E-F).

Finally, the second LI origin ARS316 did not display changes in replication timing between WT and CRE mutant cells (Figure 1F). The largest number of MATAC-Seq reads in wildtype cells corresponded to clusters 4 and 5 containing an open ARS flanked by a heterogenous accessibility profile on both sides of the origin (Figure 8H), suggesting a ‘fuzzier’ distribution of nucleosomes around this origin. These two clusters were even further enriched in the CRE mutant samples at the cost of clusters 2 and 3 that showed low accessibility and thus a more protected chromatin state on both sides of the origin. Together, this result provided further support that the combination of an open ARS flanked by well positioned nucleosomes particularly at the +1 and +2 NS is a chromatin state that supports or at least highly correlates with early and efficient replication of these four origins. Together, our MATAC-Seq data allowed us to extract such chromatin features relevant to stochastic origin activation in the four investigated replication origins that were previously masked in common population-based techniques.

## Discussion

To date, the chromatin landscape of EE and LI origins have mostly been investigated by bulk assays showing that ARS are located in a nucleosome-free region surrounded by well-positioned nucleosomes. Here, we developed MATAC-Seq as a single-molecule method combining the enrichment of targeted loci with a chromatin accessibility profiling assay, thereby providing locus-specific chromatin occupancy maps at unprecedented resolution and coverage. This provides a solid basis for detailed characterization of alternative and rare chromatin states at a given locus of interest, addressing current limitations of genome-wide single-molecule techniques to study chromatin accessibility (53,54,63,64).

Evaluation and comparison of MATAC-Seq with complementary methods is an essential step to validate our results. Averaging the single-copy locus methylation profiles could recapitulate bulk ChIP-Exo profiles and clearly identifies NFRs of replication origins as well as neighboring gene promoters (Figures 5–8). Additionally, we evaluated the MATAC-Seq profiles with an alternative single-molecule method by psoralen-crosslinking EM analysis on the multi-copy rARS locus (Supplementary Figure S5F). Since this approach requires specialized equipment as well as high input amount of DNA that we only obtained from a multi-copy gene locus, this strengthens the usefulness and feasibility of MATAC-Seq to monitor targeted single-copy domains. However, the method is currently limited to a handful of selected loci, for which genetic manipulations to insert RS/LEXA sites are required and therefore restrict the genome-wide throughput of the method. Extending MATAC-Seq by targeting and enriching larger chromosomal regions of several 10 kb –100 kb combined with the advantage of third generation long-read sequencing technologies is a future direction to be explored.

MATAC-Seq provides the first investigation of EE and LI origins after their *ex vivo* isolation from native chromosomes. Interestingly, our results show substantial level of heterogeneity at all origins investigated. There are molecules at both EE origins consistent with a fully nucleosomal configuration, but also subpopulations completely accessible without nucleosomes as well as intermediate states with well-defined surrounding chromatin structure (Figures 5–8). How such high levels of heterogeneity functionally impact the stochastic activation of replication origins is an important question. To this end, we applied MATAC-Seq to CRE mutant strains of ISW2 and INO80, implicated in nucleosome sliding and eviction (65–67). The use of genetic knock-out strains may cause indirect effects such as changes in gene expression that can cause the observed changes in chromatin accessibility as a secondary response. Although we cannot rule out this possibility, previous studies showed that a double deletion mutant of ISW2 and INO80 does not show significant differences in transcription (30,68). In addition, a genome-wide comparison of nucleosome positioning changes in an isw2Δ and an AID-tagged Isw2 degron strain both displayed a mild increase in NFR size, consistent with our observations at the Tvs1 gene promoter next to the ARS315 origin ((69) and Figure 7B). Interestingly, both isw2Δ and ies6Δ strains showed decreased replication efficiency at the EE origins (Figure 1C, D) which was accompanied with higher chromatin accessibility at the ARS (Figure 7A, B), suggesting that these chromatin remodelers are at least partially responsible to establish a regular nucleosomal pattern. This result is fully consistent with a recent study that implicated both INO80 and ISW2 CRE to cooperate with ORC in the regular nucleosome organization around origins (23). Our results additionally show that the accessibility of the NFR in collaboration with individual nucleosome positions is an important parameter at the four origins to become an efficient substrate for replication initiation (Figure 7A, B). In contrast, the accessibility of the LI origin ARS316 was also strongly increased in both mutants (Figure 7D) without apparent consequences to replication efficiency (Figure 1F). This suggests that a general increase of accessibility/loss of chromatin structure is not sufficient for efficient origin activation, but rather the presence of key neighboring chromatin components at specific locations determines replication efficiency.

Hierarchical clustering analysis revealed a large heterogeneity of chromatin states for each molecule and origin that has not been accessible for investigation (Figure 8). For consistency, we classified and divided the molecules for each origin into 5 distinct clusters that represent different chromatin states. This choice was instructed by the fact that the resulting 5 clusters explained the majority of variation in the data (Supplementary Figure S8A-D) and did not show major biases in their contribution from biological replicates (Supplementary Figure S8E) and thus likely represent biological and not technical differences in chromatin accessibility. However, there are clear examples of individual clusters that show two populations of distinct methylation states at specific regions (e.g the +3 NS region in cluster 3 of ARS315 or the +2 NS in cluster 2 of ARS313). Thus, we cannot exclude the presence of additional biologically meaningful subclusters in the data and further refinement of this analysis may provide more insights into the variability of origin chromatin. Nevertheless, the choice of 5 clusters per origin revealed clear differences in the relative contribution of reads from wildtype and CRE mutant cells (Figure 8E-H). Comparing the four origin datasets, an interesting finding is that the presence of an accessible ARS combined with well-positioned +1 and +2 NS may represent a critical parameter for efficient origin activation due to the fact that in wildtype cells, the two LI origins showed either a large fraction of molecules without an accessible ARS at ARS313 or a heterogenous nucleosomal landscape surrounding the ARS at ARS316 (Figures 7C, D and 8 G, H). The mechanistic link of how such an asymmetric chromatin state could favor the bidirectional and thus symmetric process of replication initiation is an open question, but one plausible explanation could be that the regularity of the +1 and +2 nucleosome next to the open ARS with loaded pre-RC complexes provides a beneficial chromatin substrate in the right distance and orientation for the recruitment or binding of additional factors, for example the replication initiation factors Cdc45, Sld2 or Sld3 known to be limiting for early and efficient origin firing (70). Interestingly, the INO80 CRE was previously shown to co-localize with the origin recognition complex (ORC) at yeast replication origins to prevent pervasive transcription through EE origin sequences (71). As the process of transcription is mostly unidirectional, it is tempting to speculate that the observed bias in reduced nucleosome occupancy on one side of the origin could be functionally related to the occurrence of cryptic transcription processes. To monitor the enrichment of transcription-associated factors, an interesting perspective would be to perform proteomic analysis of the purified chromatin domains in the CRE mutant strains, as we have recently established in the wildtype condition (45).

One complexity in interpreting our MATAC-Seq data is the fact that besides nucleosome core particles also other protein complexes bound to the native chromatin domains can contribute to the differential accessibilities and observed heterogeneity. Indeed, our published mass spectrometry study of the four origins showed a clear enrichment of all subunits of the MCM2-7 pre-RC complex on the purified chromatin domains (45), suggesting that loaded MCM2-7 double hexamers may likely contribute to the observed accessibilities. Our initial attempts to identify a characteristic ∼50–60 bp MCM2-7 double hexamer footprint were unsuccessful (data not shown), which can be explained by several technical and/or biological reasons. First, we cannot exclude the possibility that other factors and protein complexes such as ORC, CRE, transcription factors or RNA polymerase complexes may contribute to the observed DNA footprints on single DNA molecules. Second, in case of a nucleosome being positioned in close proximity to the pre-RC complex, it is expected that this would result in a larger footprint in the size range of ∼190–220bp and would thus not be detectable as an MCM2–7 footprint. Third, several studies have shown that multiple MCM2–7 complexes can be loaded per origin and once loaded, MCM2–7 double hexamers may slide away from origins, allowing repeated loading. Although the efficiency of our DNA methylation reaction has been optimized to reach saturation within a short incubation time of 4 × 7.5 min, we cannot exclude the possibility that some of the MCM2–7 double hexamers can slide along DNA during the reaction time and therefore interfere with precise footprinting of this complex. Finally, a technological limitation is that the DNA methylation signal does not achieve single-nucleotide resolution and depends on the DNA sequence context. To illustrate this, we compared the local variation of the Nanopore signal in a base-by-base manner between a fully methylated and unmethylated plasmid DNA control and observed a high variation of read scores depending on genomic position and sequence context (Supplementary Figure S8J). Thus, having multiple consecutive methylation sites is essential to account for this variation and reach a reliable footprint. In the future, more advanced methylation calling algorithms could improve the accuracy across different DNA sequence contexts, thereby opening the possibility of detecting the occupancy of smaller protein complexes and even transcription factors on a single molecule basis.

Importantly, our cutoffs for methylation calling (1 for m6a and 2 for CpG, see red dashed lines in Supplementary Figure S8J) are extremely conservative, thereby making sure that no methylated bases are called in the unmethylated control sample. Although our stringent cut-offs will likely result in false-negative methylation callings, we want to stress that the observed local variation of methylation calling is currently an inherent limitation of the technology and lowering this threshold is likely introducing an additional source of technical noise and therefore increasing the heterogeneity due to technical but not biological reasons. It is crucial to highlight that our analysis is not rooted in comparative evaluations between different sites or origins. Our focus remains exclusively on contrasting changes between wildtype and CRE mutant cells within the same DNA sequence context. Given that the thresholding errors are systematic and consistent across different conditions of the same origin, we believe our interpretations remain robust and unaffected by this limitation.

Clearly, the small number of replication origins in this study is another limitation that restricts our ability to draw global and generalized conclusions on the critical features of origin chromatin towards efficient replication activation beyond the investigated loci. Nevertheless, the native chromatin purification strategy combined with MATAC-Seq gives us the opportunity to strongly correlate and further dissect the functional relationship between the chromatin accessibility landscape and the replication profiles of these four ARS loci and represents a first step towards our goal to link the observed changes in chromatin state and replication efficiency of the locus. In future work, we intend to further explore this connection by using native chromatin templates as substrates for controlled *in vitro* replication assays to unravel the precise role of chromatin in origin regulation and function. Thus, we expect MATAC-Seq as a broadly applicable long-read single-molecule method to study the functional importance of heterogeneity of chromatin states, which is a major driver for cellular plasticity implicated in development as well as many human diseases such as cancer (72–77).

## Supplementary Material

gkad1022_Supplemental_File

## Data Availability

The raw sequencing data underlying this article are available at the Gene Expression Omnibus at https://www.ncbi.nlm.nih.gov/geo/ under accession number GSE220757. Scripts and the binary count matrices are available in Zenodo at https://doi.org/10.5281/zenodo.10018261.
