# Supplementary material for: Single molecule MATAC-seq reveals key determinants of DNA replication origin efficiency
Source: Nucleic Acids Res. 2023 Nov 13;51(22):12303–24. doi: 10.1093/nar/gkad1022 (PMC10711542; doi:10.1093/nar/gkad1022)
Supplement: gkad1022_Supplemental_File [file gkad1022_supplemental_file.docx]

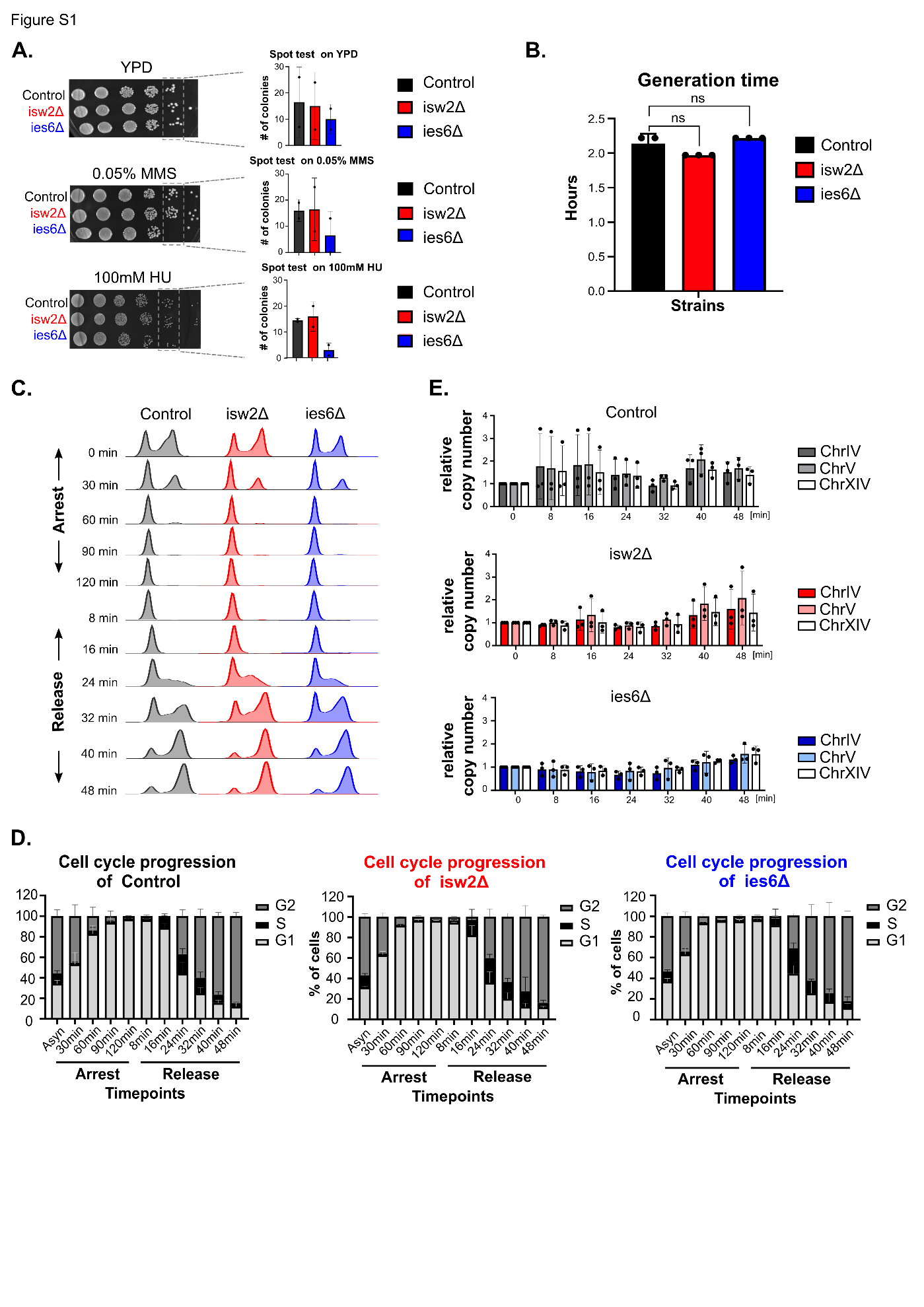
**Supplementary Figures**

**Figure S1.**  **A.** Spot test of WT (Y66 - control) and CRE mutant (Y104 - isw2Δ and Y136 - dies6Δ) strains. The cells were grown on YPD medium or on YPD medium in the presence of 100mM hydroxyurea (HU) or 0.05% Methyl methanesulfonate (MMS). The plots show the number of obtained single yeast colonies at the 10^4^ serial dilution (box with dashed lines) after growth at 30°C for three days. **B.** Generation timing of Y66 (control), Y104 (isw2Δ) and Y136 (dies6Δ). The strains were grown in YPD medium and OD600 measurements were taken every 360 sec by a multi-plate reader to determine the generation time of the yeast strains. **C.** Cell cycle progression of control and mutant strains. The cells were arrested by 50 ng/ml α-factor and released into S-phase by addition of 125U pronase. The FACS samples were taken at the indicated timepoints and side-by-side with the DNA samples subjected to qPCR analysis in **Fig.1C-F**. **D.** FACS quantification of the distribution of G1, S and G2 phase cells in arrest and release timepoints of Y66 (control), Y104 (isw2Δ) and Y136 (dies6Δ) strains. **E.** Comparative analysis of relative copy numbers calculated from the Ct values obtained from the late replicating regions of ChrIV, ChrV and ChrXIV. The relative copy number values were calculated from the replication timing qPCR experiments using the strains Y66 (control), Y104 (isw2Δ) and Y136 (dies6Δ) and the formula 1/2^Ct. The resulting values were normalized to the 0 [min] G1 arrest sample and relative copy number changes plotted over the S-phase release timecourse. Average and standard deviations in all graphs of Fig. S1 are from n= 3 biological replicates.


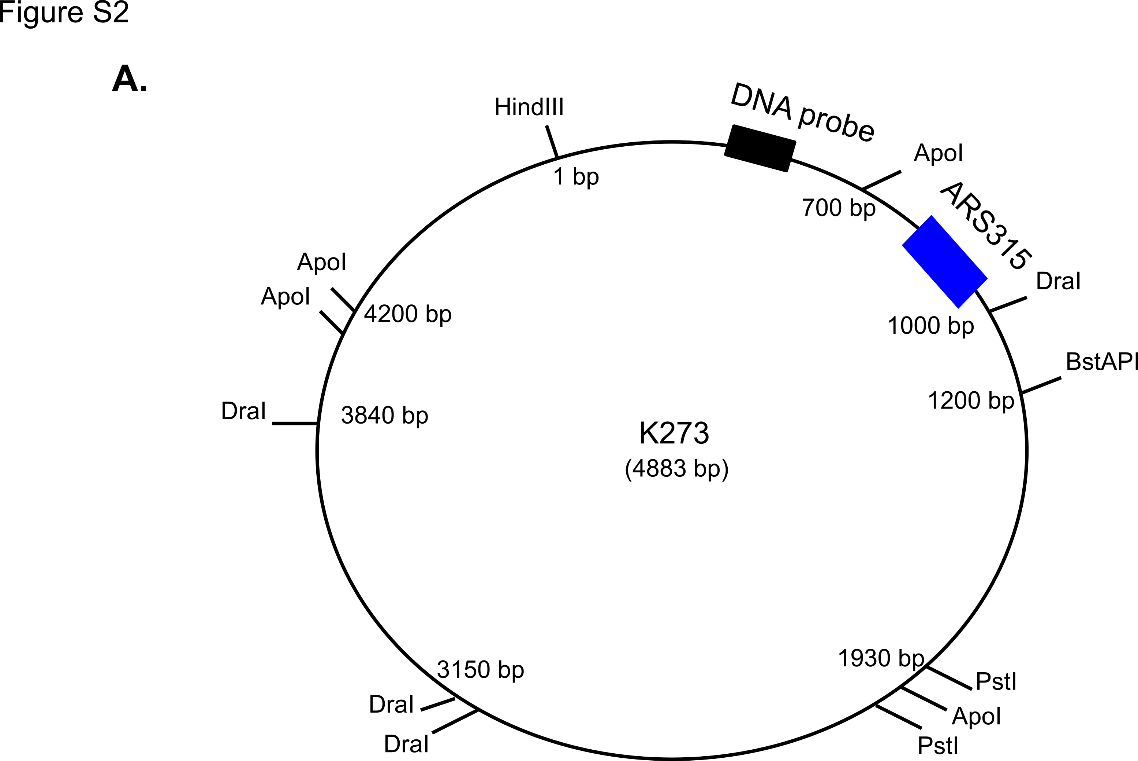


**Figure S2. A.** Schematic presentation of plasmid K273 containing the DNA sequence of ARS315 locus used as spike in control of the REA analysis of ies6Δ-ARS315 strains. The black rectangle indicates the position of the DNA probe used for the Southern blot and the blue rectangle indicates the position of the ARS315 locus. On the map it is shown the distance between the restriction sites using the unique HindIII restriction site as the starting point. Similar to the native chromatin locus, the plasmid was initially digested by BstAPI, DraI and ApoI, separately, and later all samples were digested by HindIII and PstI (secondary digestion). The expected detectable DNA fragments according to the two digestion steps and the position of the probe are 1200 bp (for BstAPI and HindIII), 1000bp (for DraI and HindIII) and 700bp (for ApoI and HindIII). The larger fragments 4110 bp (in BstAPI) and 1300bp (in ApoI) indicate inefficient digestion of HindIII. All the fragments deriving from the plasmid are noted by asterisks in **Fig. 2D**.


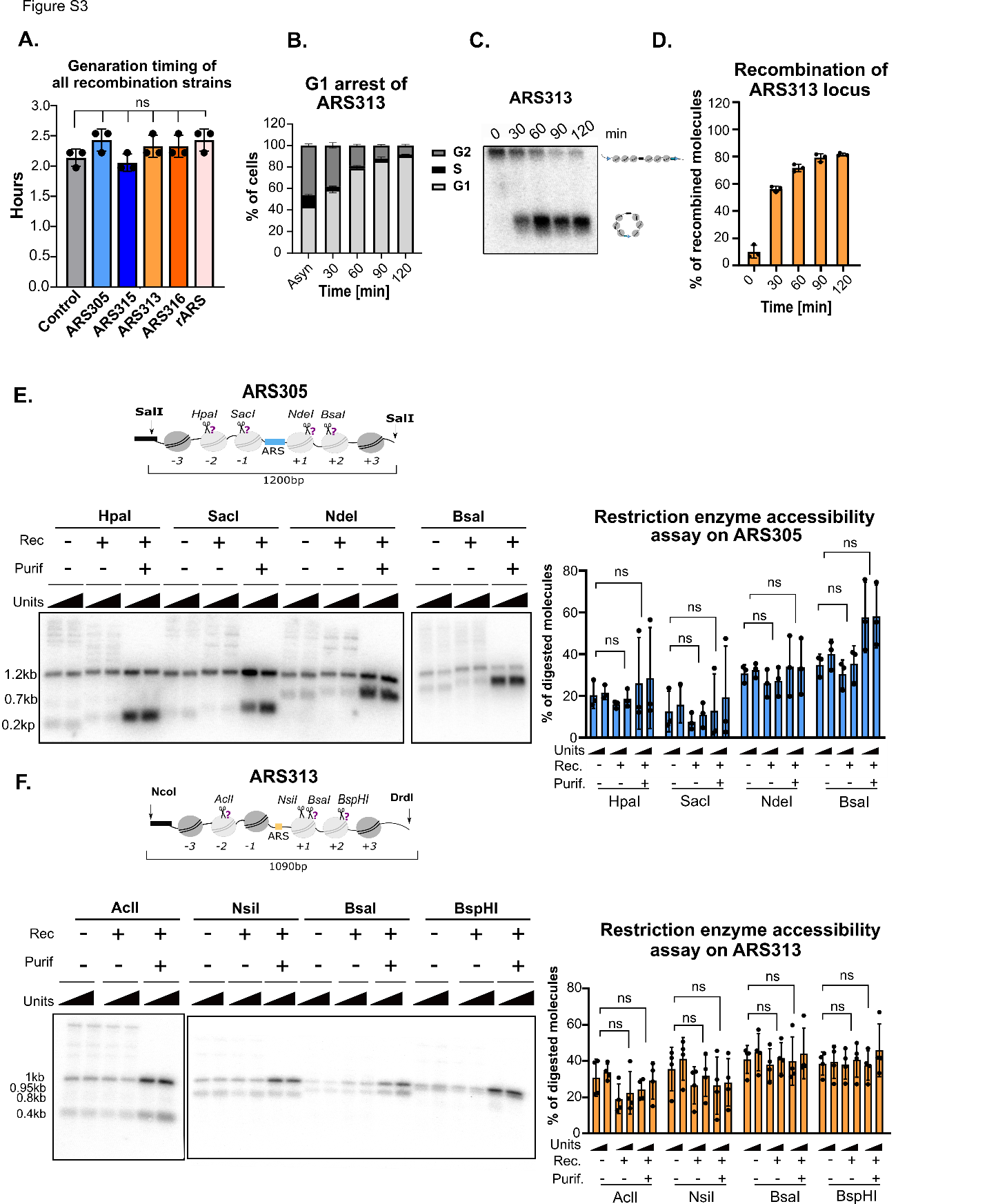


**Figure S3. A.** Generation timing of yeast strains competent for recombination (Y65-ARS305, Y91-ARS315, Y94-ARS313, Y69-ARS316, Y84-rARS) and control (Y66-without RS/LexA). The strains were grown in YPD medium and OD600 measurements were taken every 360 sec by a multi-plate reader. **B.** Cell arrest in G1 phase. The strain Y94-ARS313 was grown in YPR medium and arrested with 50 ng/ml α-factor. Samples for FACS analysis were taken at the indicated timepoints and stained by Sytox green to monitor the distribution of G1, S and G2 phase in both profiles. **C.** Recombination kinetics of ARS313 loci. The strain Y94-ARS313 and Y66-control (see **Fig.3C**) were grown in YPR medium to logarithmic phase and arrested in G1 phase by addition of α-factor (50ng/ml) and recombination was induced by the addition of 2% galactose. Samples were taken at indicated timepoints. DNA was isolated and linearized by BsrGI (in Y66 **Fig.3C**) and by NcoI (in Y94) and subjected to Southern blot analysis. The positions of unrecombined and recombined molecules are shown on the right. **D.** The histogram shows the results of Southern blot as percentage of recombined chromatin locus. **E-F.** Comparative REA analysis in chromosomal, recombined and purified ARS305 and ARS313 locus. Nuclei (before and after recombination) and chromatin rings (after purification) from yeast strains Y65 (ARS305), Y94 (ARS313) were isolated and digested with increasing amounts of the indicated restriction enzymes (scissors). DNA was isolated, digested with SalI in ARS305, NcoI / DrdI in ARS313 and subjected to indirect end-labeling Southern blot analysis with the radioactively labeled probe. The histogram shows the results of Southern blot quantification as a percentage of digested chromatin locus. Mean and standard deviations of all plots in **Fig.S3** are from n= 3 or 4 biological replicates (ns, indicates no significant statistical difference P > 0.05, unpaired t-test).


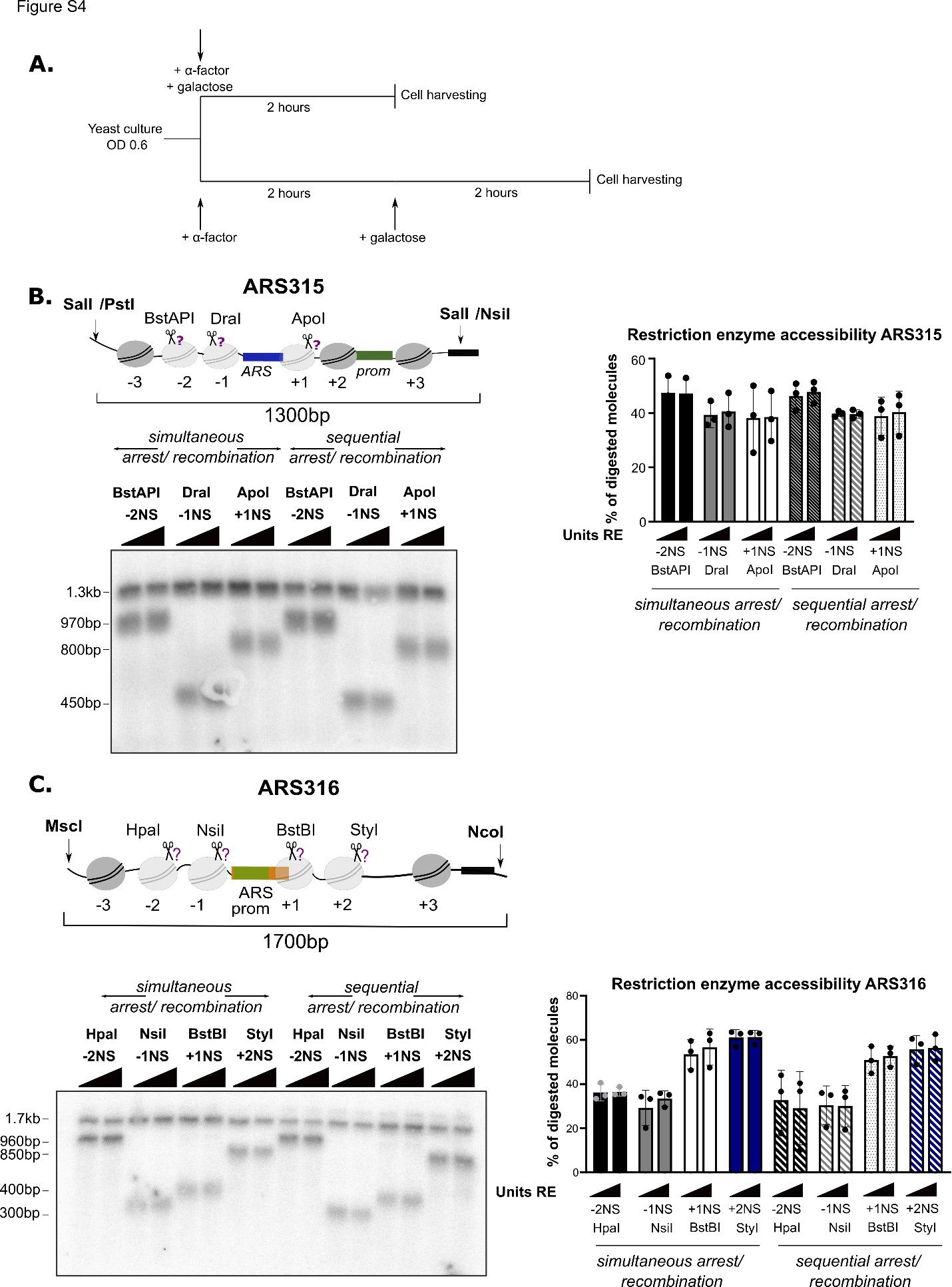


**Figure S4. A.** Experimental outline for comparing the chromatin accessibility of a specific locus after simultaneous or sequential cell cycle arrest and site-specific recombination reaction. **B-C.** Comparative REA analysis in chromosomal recombined ARS315 and ARS316 locus, which have been either arrested and recombined simultaneously or sequentially. Nuclei from yeast strains Y91 (ARS315), Y69 (ARS316) were isolated and digested with increasing amounts of the indicated restriction enzymes (scissors). DNA was isolated, digested with SalI/PstI in ARS315, MscI / NcoI in ARS316 and subjected to indirect end-labeling Southern blot analysis with the radioactively labeled probe. The histogram shows the results of Southern blot quantification as a percentage of digested chromatin locus. Mean and standard deviations of all plots in **Fig.S4** are from n= 3 biological replicates.


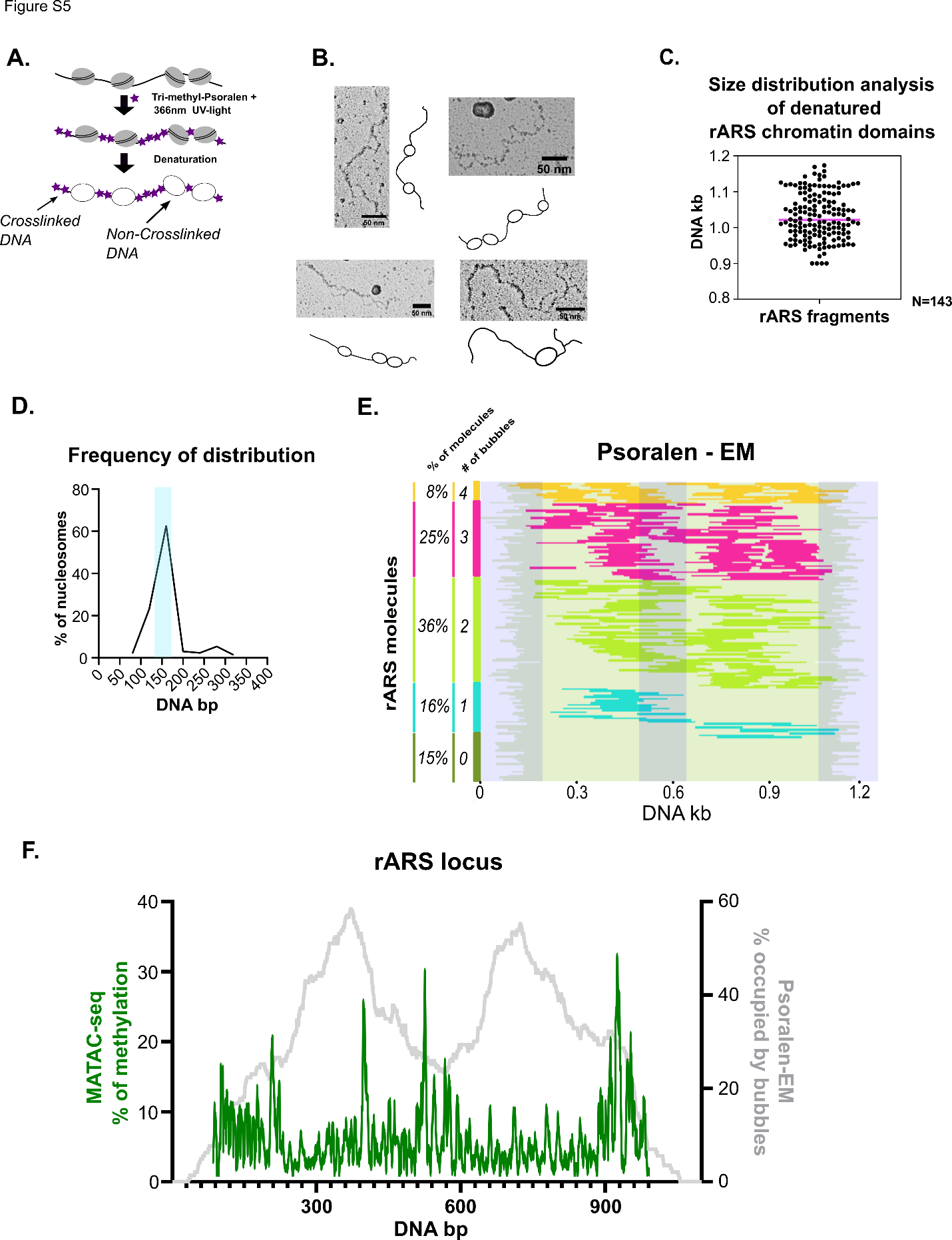


**Figure S5. A.** Schematic representation of the principal of the psoralen crosslinking assay. **B.** Determination of nucleosome positions at the rARS locus by single molecule EM analysis. Purified rARS chromatin rings were psoralen-crosslinked. After DNA isolation, the rings were digested by NcoI and then subjected to denaturing spreading followed by EM analysis. 143 molecules were analyzed by measuring the size and the number of each nucleosomal bubble. Representative electron micrographs for molecules containing different numbers of nucleosomes are shown on the left. **C.** Size distribution of the purified and denatured rARS fragments as analyzed by electron microscopy. The expected length of the rARS locus is 1046 bp +/-10% The pink line indicates the median. **D.** The plot shows the size distribution of the nucleosomal bubbles. The expected nucleosomal size is indicated in light blue. **E.** Five different groups of rARS molecules are depicted according to the number of nucleosomes (0, 1, 2, 3 or 4) and its coverage amongst the whole population. Each molecule has been aligned to its symmetric center position. The shadowed areas indicate the middle and the two ends of the rARS molecules. **F.** Averaging nucleosome profile of 143 unoriented rARS molecules. The plot shows the probability of a nucleosome in a specific locus capturing the less protected rARS origin and the two most prominent neighboring nucleosomal sites which is comparable to the averaging methylation profile derived from MATAC-seq.


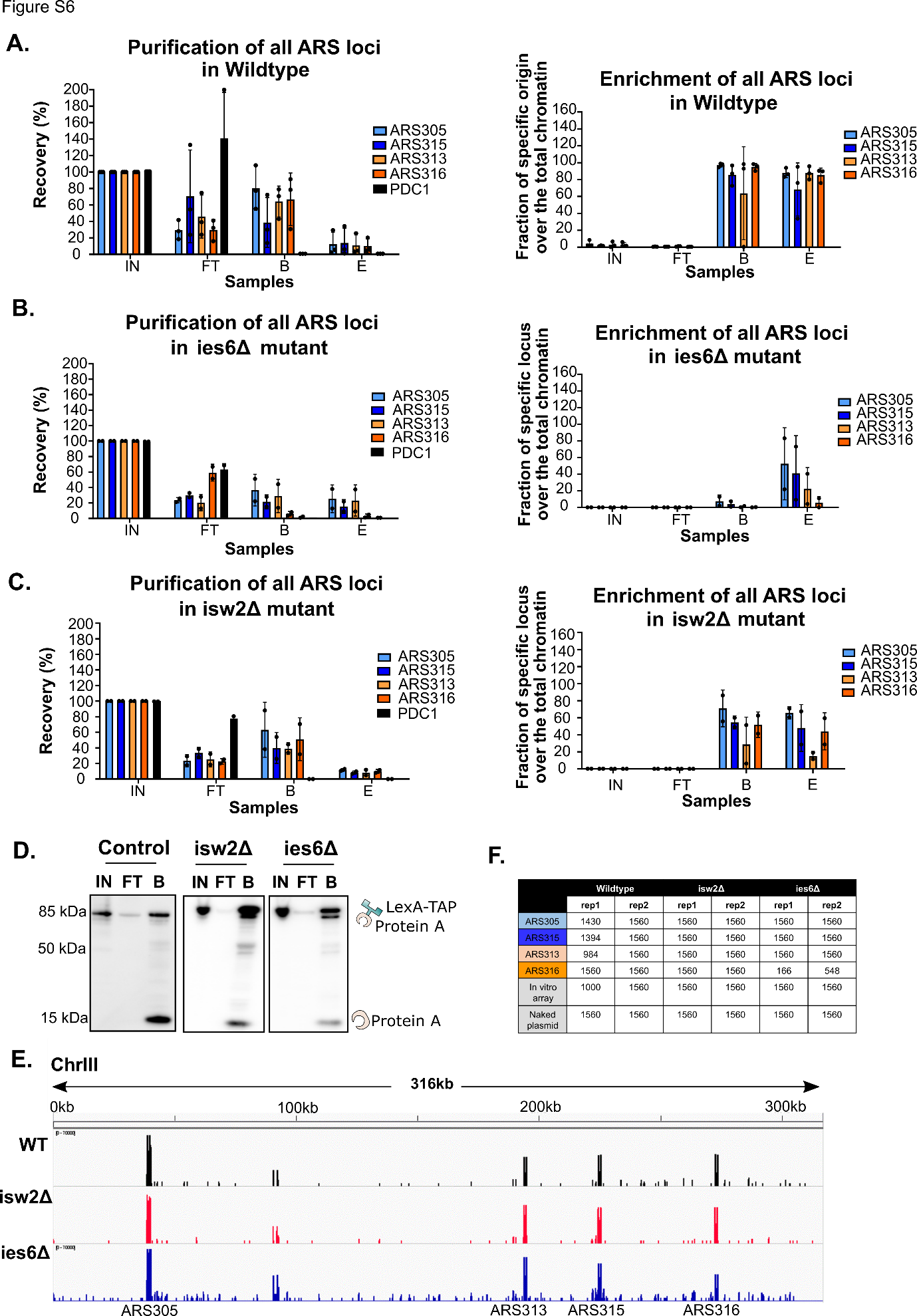


**Figure S6.** **A**. LexA affinity purification process was performed for all the WT strains together, which were then subjected to MATAC-Seq assay. DNA samples were taken (0.1% IN, FT, B, E) from n=2 analyzed by qPCR. The enrichment of an unrelated region (PDC1) was tracked side-by-side with the origin regions of interest. The plot on the left shows how much of the total DNA was originated by the chromatin locus of interest. Given that the size of yeast genome is 12 Mbp, and the length of a chromatin ring is ~1 kb, the fold enrichment ratio of the specific origin to PDC1 was used to define the enrichment of an origin in the DNA samples. **B-C**. The same strategy was used to analyze the DNA samples of the CRE mutant strains (ies6Δ and isw2Δ). **D.** Protein analysis of LexA affinity purification process. The protein samples (0,5% IN, FT, B, E) were collected in parallel with the previous DNA samples and loaded on an SDS gel in order to monitor the LexA protein during the purification by Western blot. Samples were taken from WT, ies6Δ and isw2Δ. **E.** Enrichment of the targeted purified loci on ChrIII after MATAC-seq performance. **F.** Table indicating the number of reads per replicate and condition that have been used for MATAC-Seq analysis.


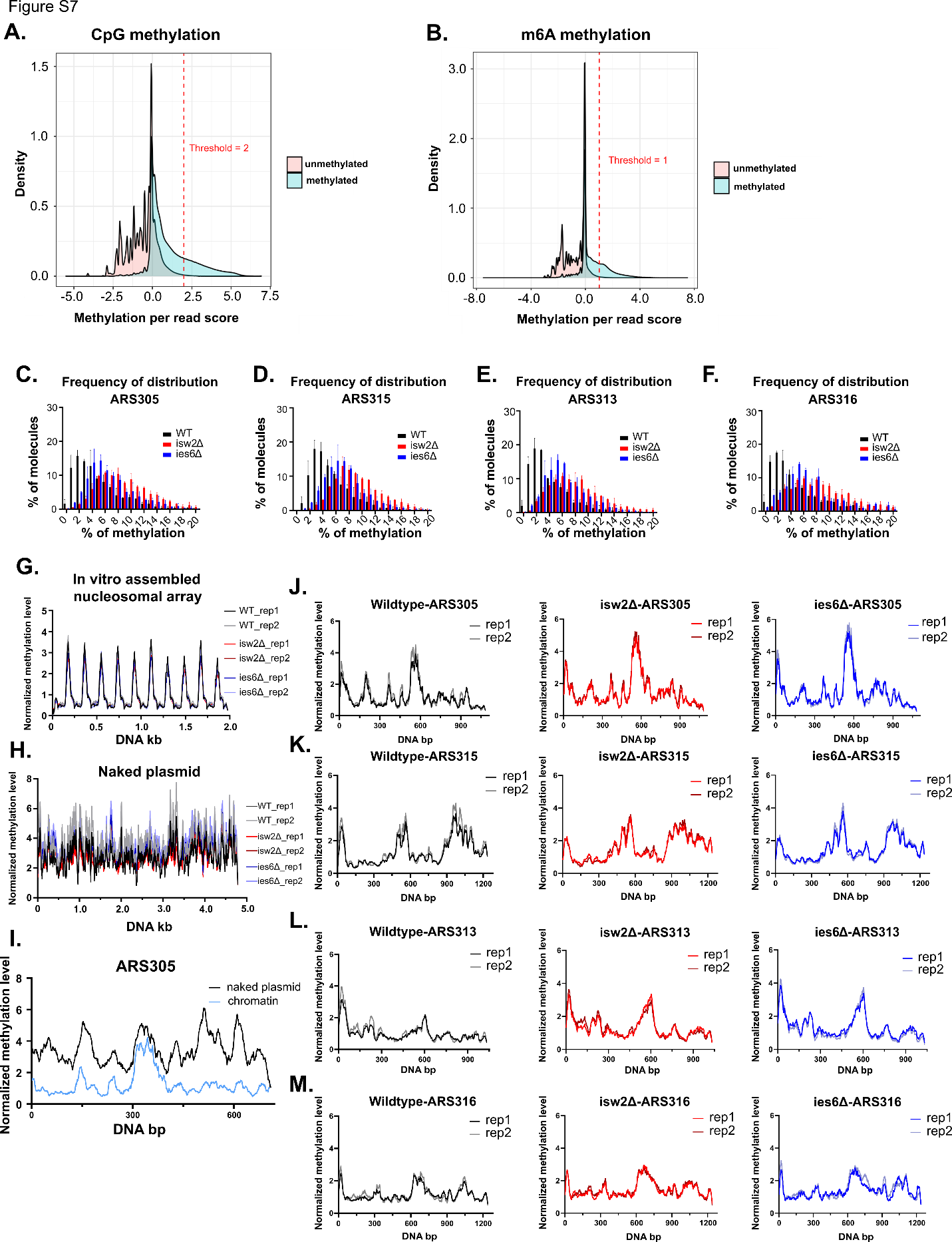


**Figure S7. A-B.** Methylation density comparison between a CpG/m6A methylated and an unmethylated plasmid. The dashed line indicates the threshold that was used to distinguish between signal and noise of methylation on the Nanopore sequencing data. **C-F.** Frequency of methylation distribution. Comparative analysis of frequency of methylation distribution in all replication origins between WT and CRE mutants shows an overall increase of unprotected regions in CRE mutants. Average and standard deviations are from n= 2 biological replicates. **G-H.** The plots show the methylation pattern on the *in vitro* nucleosomal array and naked plasmid (controls) of both biological replicates after normalization to maximum methylation. **I.** Comparative analysis of the same DNA sequence on ARS305 between naked plasmid and chromatin. The unprotected ARS domain of chromatin shows similar level of accessibility with the naked DNA. Their methylation level differs on the surrounding nucleosomal regions. **J-M.** Methylation pattern on native chromatin domains at ARS loci for both biological replicates. For all plots in **Fig. S7**, the methylation level has been normalized to the average methylation of the nucleosomal array of the respective replicate and smoothened using a 30bp window.


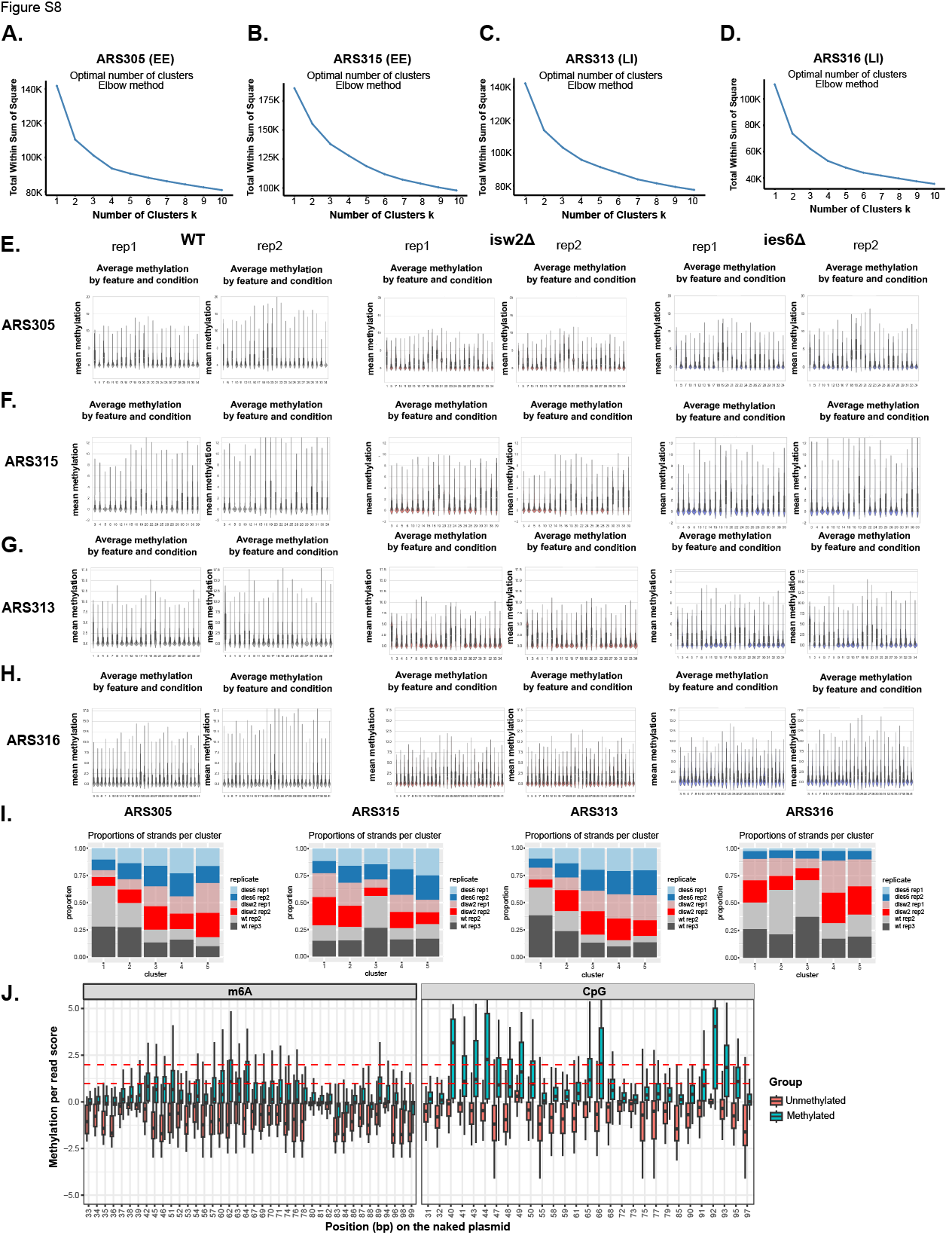


**Figure S8 A-D.** Elbow plots that display on the y-axis the Within-Cluster-Sum of Square (WSS) against the different number of clusters (k) on the x-axis for each of the four origin datasets. **E-H.** Average mean methylation of each of the 30bp genomic bins showing statistical significance along each chromatin domain of both biological replicates in WT, isw2Δ and ies6Δ strains. **I.** The plots show the read distribution of wildtype and CRE mutants per cluster and biological replicates. **J.** Local variation of Megalodon-based m6A (left) and CpG (right) methylation calling between an unmethylated (ctr) and a fully methylated (met) control plasmid. Each plot shows the variation in the methylation per read score along a stretch of 100 bp of the plasmid DNA based on 3000 independent single-molecule reads. Dashed lines indicate the used cutoffs 1 and 2 for m6a and CpG methylation, respectively.

**Supplementary Tables**

**Supplementary Table S1: Yeast strains used in this study.**

| **ID** | **Genotype** | **Comment** | **Cloning Strategy** |
| --- | --- | --- | --- |
| Y0001 (Y01408) | MATa; ura3Δ0; leu2Δ0; his3Δ1; met15Δ0; bar1::kanMX4 | Wildtype strain | EUROSCARF |
| Y0008 (ySH089) | MATa; ade2-1; ura3-1; trp1-1; leu2-3,112; his3-11; can1-100 | URA3::LEU2 pTEF2 LEXA-TAP pGAL RecR | Transformation of SfbI digested fragment from plasmid pSH21 |
| Y0010 (yMW2) | MATa; ura3Δ0; leu2Δ0; his3Δ1; met15Δ0; bar1::kanMX4; ARS305::URA3 | ARS305 exchanged for URA3 | Transformation of amplicon derived from K001 (primer 0040/0041) into Y0001. Selection on Ura |
| Y0011 (yMW3) | MATa; ura3Δ0; leu2Δ0; his3Δ1; met15Δ0; bar1::kanMX4; ARS316::URA3 | ARS316 exchanged for URA3 | Transformation of amplicon derived from K001 (primer 0080/0081) into Y0001. Selection on Ura |
| Y0018 (yMW6) | MATa; ura3Δ0; leu2Δ0; his3Δ1; met15Δ0; bar1::kanMX4; RS_LEXA_NS-3_ARS305_NS+3_RS | RS sites and lexA binding sites at ARS305 after +-3 nucleosomes | Transformation of amplicon derived from K113 (primer 0223/0224) into Y0010. Selection on FOA |
| Y0021 (yMW9) | MATa; ura3Δ0; leu2Δ0; his3Δ1; met15Δ0; bar1::kanMX4; RS_LEXA_NS-3_ARS316_NS+3_RS | RS sites and lexA binding sites at ARS316 after +-3 nucleosomes | Transformation of amplicon derived from K116 (primer 0251/0252) into Y0011. Selection on FOA |
| Y0042 (yAC02) | MATa; ura3Δ0; leu2Δ0; his3Δ1; met15Δ0; bar1::kanMX4; ARS313::URA3 | ARS313 exchanged for URA3 | Transformation of amplicon derived from K001 (primer 0338/0457) into Y0001. Selection on Ura |
| Y0043 (yAC03) | MATa; ura3Δ0; leu2Δ0; his3Δ1; met15Δ0; bar1::kanMX4; ARS315::URA3 | ARS315 exchanged for URA3 | Transformation of amplicon derived from K001 (primer 0340/0458) into Y0001. Selection on Ura |
| Y0044 (yAC04) | MATa; ura3Δ0; leu2Δ0; his3Δ1; met15Δ0; bar1::kanMX4;RS_LEXA_NS-3_ARS313_NS+3_RS | RS sites and lexA binding sites at ARS313 after +-3 nucleosomes | Transformation of EcoRI/HindIII digested plasmid K170 into Y0042. Selection on FOA |
| Y0065 (yTS3) | MATa; ura3Δ0; leu2Δ0; his3Δ1; met15Δ0; bar1::kanMX4; RS_LEXA_NS-3_ARS305_NS+3_RS; Chr I 212kb::LEU2 pTEF2-LEXA-TAP pGAL1-10 RecR | RS sites and lexA binding sites at ARS305 after +-3 nucleosomes. Expression cassette for R Recombinase and lexA (TEF2 promoter) | Transformation of SbfI digested plasmid K238 into Y0018. Selection on Leu |
| Y0066 (yTS4) | MATa; ura3Δ0; leu2Δ0; his3Δ1; met15Δ0; bar1::kanMX4; Chr I 212kb::LEU2 pTEF2-LEXA-TAP pGAL1-10 RecR | WT strain with expression cassette for R Recombinase and lexA (TEF2 promoter) | Transformation of SbfI digested plasmid K238 into Y0001. Selection on Leu |
| Y0069 (yMW38) | MATa; ura3Δ0; leu2Δ0; his3Δ1; met15Δ0; bar1::kanMX4; RS_LEXA_NS-3_ARS316_NS+3_RS; Chr I 212kb::LEU2 pTEF2-LEXA-TAP pGAL1-10 RecR | RS sites and lexA binding sites at ARS316 after +-3 nucleosomes. Expression cassette for R Recombinase and lexA (TEF2 promoter) | Transformation of SbfI digested plasmid K238 into Y0021. Selection on Leu |
| Y0084 (yMW53) | MATa; ade2-1; ura3-1; trp1-1; leu2-3,112; his3-11; can1-100; bar1::his3 | Bar1 knockout | Transformation of amplicon derived from K207 (primers 932/933) into Y8. Selection on His |
| Y0089 (yAC14) | MATa; ura3Δ0; leu2Δ0; his3Δ1; met15Δ0; bar1::kanMX4;RS_LEXA_NS-3_ARS315_NS+3_RS | RS sites and lexA binding sites at ARS315 after +-3 nucleosomes | Transformation of amplicon derived from K273 (primer 0858/0859) into Y0043. Selection on FOA |
| Y0091 (yAC16) | MATa; ura3Δ0; leu2Δ0; his3Δ1; met15Δ0; bar1::kanMX4;RS_LEXA_NS-3_ARS315_NS+3_RS; Chr I 212kb::LEU2 pTEF2-LEXA-TAP pGAL1-10 RecR | RS sites and lexA binding sites at ARS315 after +-3 nucleosomes. Expression cassette for R Recombinase and lexA (TEF2 promoter) | Transformation of SbfI digested plasmid K238 into Y0089. Selection on Leu |
| Y0094 (yAC19) | MATa; ura3Δ0; leu2Δ0; his3Δ1; met15Δ0; bar1::kanMX4;RS_LEXA_NS-3_ARS313_NS+3_RS; Chr I 212kb::LEU2 pTEF2-LEXA-TAP pGAL1-10 RecR | RS sites and lexA binding sites at ARS313 after +-3 nucleosomes. Expression cassette for R Recombinase and lexA (TEF2 promoter) | Transformation of SbfI digested plasmid K238 into Y0044. Selection on Leu |
| Y0102 (yAC20) | MATa; ura3Δ0; leu2Δ0; his3Δ1; met15Δ0; bar1::kanMX4;RS_LEXA_NS-3_ARS315_NS+3_RS; Chr I 212kb::LEU2 pTEF2-LEXA-TAP pGAL1-10 RecR ; isw2Δ::ura3 | Deletion of isw2 gene | Transformation of amplicon derived from K1 (primers 848/849) into Y0091. Selection on URA |
| Y0103 (yAC21) | MATa; ura3Δ0; leu2Δ0; his3Δ1; met15Δ0; bar1::kanMX4;isw2Δ::ura3 | Deletion of isw2 gene | Transformation of amplicon derived from K1 (primers 848/849) into Y0001. Selection on URA |
| Y0104 (yAC22) | MATa; ura3Δ0; leu2Δ0; his3Δ1; met15Δ0; bar1::kanMX4; Chr I 212kb::LEU2 pTEF2-LEXA-TAP pGAL1-10 RecR ;isw2Δ::ura3 | Deletion of isw2 gene | Transformation of amplicon derived from K1 (primers 848/849) into Y0066. Selection on URA |
| Y0105 (yAC23) | MATa; ura3Δ0; leu2Δ0; his3Δ1; met15Δ0; bar1::kanMX4; RS_LEXA_NS-3_ARS305_NS+3_RS; Chr I 212kb::LEU2 pTEF2-LEXA-TAP pGAL1-10 RecR ;isw2Δ::ura3 | Deletion of isw2 gene | Transformation of amplicon derived from K1 (primers 848/849) into Y0065. Selection on URA |
| Y0106 (yAC24) | MATa; ura3Δ0; leu2Δ0; his3Δ1; met15Δ0; bar1::kanMX4; RS_LEXA_NS-3_ARS316_NS+3_RS; Chr I 212kb::LEU2 pTEF2-LEXA-TAP pGAL1-10 RecR;isw2Δ::ura3 | Deletion of isw2 gene | Transformation of amplicon derived from K1 (primers 848/849) into Y0069. Selection on URA |
| Y0107 (yAC25) | MATa; ura3Δ0; leu2Δ0; his3Δ1; met15Δ0; bar1::kanMX4;RS_LEXA_NS-3_ARS313_NS+3_RS; Chr I 212kb::LEU2 pTEF2-LEXA-TAP pGAL1-10 RecR;isw2Δ::ura3 | Deletion of isw2 gene | Transformation of amplicon derived from K1 (primers 848/849) into Y0069. Selection on URA |
| Y0127 (yAC37) | MATa; ura3Δ0; leu2Δ0; his3Δ1; met15Δ0; bar1::kanMX4; RS_LEXA_NS-3_ARS305_NS+3_RS; Chr I 212kb::LEU2 pTEF2-LEXA-TAP pGAL1-10 RecR ;ies6Δ::his | Deletion of ies6 gene | Transformation of SbfI and SacI digested plasmid K303 into Y0065. Selection on histidine |
| Y0128 (yAC38) | MATa; ura3Δ0; leu2Δ0; his3Δ1; met15Δ0; bar1::kanMX4;RS_LEXA_NS-3_ARS313_NS+3_RS; Chr I 212kb::LEU2 pTEF2-LEXA-TAP pGAL1-10 RecR ;ies6Δ::his | Deletion of ies6 gene | Transformation of SbfI and SacI digested plasmid K303 into Y0094. Selection on histidine |
| Y0129 (yAC39) | MATa; ura3Δ0; leu2Δ0; his3Δ1; met15Δ0; bar1::kanMX4; RS_LEXA_NS-3_ARS316_NS+3_RS; Chr I 212kb::LEU2 pTEF2-LEXA-TAP pGAL1-10 RecR;ies6Δ::his | Deletion of ies6 gene | Transformation of SbfI and SacI digested plasmid K303 into Y0069. Selection on histidine |
| Y0130 (yAC40) | MATa; ura3Δ0; leu2Δ0; his3Δ1; met15Δ0; bar1::kanMX4;RS_LEXA_NS-3_ARS315_NS+3_RS; Chr I 212kb::LEU2 pTEF2-LEXA-TAP pGAL1-10 RecR ;ies6Δ::his | Deletion of ies6 gene | Transformation of SbfI and SacI digested plasmid K303 into Y0091. Selection on histidine |
| Y0135 (yAC44) | MATa; ura3Δ0; leu2Δ0; his3Δ1; met15Δ0; bar1::kanMX4; Chr I 212kb::LEU2 pTEF2-LEXA-TAP pGAL1-10 RecR ;ies6Δ::his | Deletion of ies6 gene | Transformation of SbfI and SacI digested plasmid K303 into Y0066. Selection on histidine |
| Y0136 (yAC45) | MATa; ade2-1; ura3-1; trp1-1; leu2-3,112; his3-11; can1-100; bar1::his3; isw2Δ::ura3 | Deletion of isw2 gene | Transformation of amplicon derived from K1 (primers 848/849) into Y0084. Selection on URA |

**Supplementary Table S2: Oligonucleotide sequences used in this study**

| **ID** | **Sequence (5' to 3')** | **Application/Purpose** |
| --- | --- | --- |
| #0016 | GATAAGCTTttttgggtcctttgttttcg | Primer used for PCR together with ARS305_downstream (#0017) to amplify ARS305 replication origin in pBlueSkript using HindIII and EcoRI site adapter |
| #0017 | GATGAATTCatttggagggaggagaagga | Primer used for PCR together with ARS305_upstream (#0016) to amplify ARS305 replication origin in pBlueSkript using HindIII and EcoRI site adapter |
| #0020 | TCAGAATTCtcctccagtgggattgctac | Primer used for PCR together with ARS316_downstream (#0021) to amplify ARS316 replication origin in pBlueSkript using EcoRI and PstI site adapter |
| #0021 | TCACTGCAGgcctagcgggctattacctt | Primer used for PCR together with ARS316_upstream (#0020) to amplify ARS316 replication origin in pBlueSkript using EcoRI and PstI site adapter |
| #0040 | ttttgggtcctttgttttcgttgtttcagtctggataaattttaagttac tcgatccgatgataagctgtc | Primer used for PCR together with ARS305_URA3 rev (#0041) to amplify URA3 marker gene with homology arms for genomic deletion of ARS305 |
| #0041 | ATTTGGAGGGAGGAGAAGGATAACAGCGACGAAACACCGGACAGATTCCCtgccacctgacgtctaagaa | Primer used for PCR together with ARS305_URA3 fwd (#0040) to amplify URA3 marker gene with homology arms for genomic deletion of ARS305 |
| #0046 | GAAAGTAGTTATTACGGCGTCGG | Primer used for Gibson assembly PCR together with ARS305-2+2_G1_rev (#0047) to generate modified ARS305 locus with RS LEXA sites spanning -2 and +2 nucleosome |
| #0047 | GGCTCTAGGGTAGTTGCG | Primer used for Gibson assembly PCR together with ARS305-2+2_G1_rev (#0046) to generate modified ARS305 locus with RS LEXA sites spanning -2 and +2 nucleosome |
| #0048 | gcgacgcccgacgccgtaataactactttcTCGACCCGAGATCATATC | Primer used for Gibson assembly PCR together with ARS305-2+2_G2_rev (#0049) to generate modified ARS305 locus with RS LEXA sites spanning -2 and +2 nucleosome |
| #0049 | caatgagagaaacgcaactaccctagagccCCACGATTTGATGAAAGAATAAC | Primer used for Gibson assembly PCR together with ARS305-2+2_G2_fwd (#0048) to generate modified ARS305 locus with RS LEXA sites spanning -2 and +2 nucleosome |
| #0050 | GTGTGCTAAGTGTCCTGTTTC | Primer used for Gibson assembly PCR together with ARS305-3+3_G1_rev (#0051) to generate modified ARS305 locus with RS LEXA sites spanning -3 and +3 nucleosome |
| #0051 | AATATTGTCTTTGGACGTTTG | Primer used for Gibson assembly PCR together with ARS305-3+3_G1_rev (#0050) to generate modified ARS305 locus with RS LEXA sites spanning -3 and +3 nucleosome |
| #0052 | gaacgttccgaaacaggacacttagcacacTCGACCCGAGATCATATC | Primer used for Gibson assembly PCR together with ARS305-3+3_G2_rev (#0053) to generate modified ARS305 locus with RS LEXA sites spanning -3 and +3 nucleosome |
| #0053 | tggtttgggcaaacgtccaaagacaatattCCACGATTTGATGAAAGAATAAC | Primer used for Gibson assembly PCR together with ARS305-3+3_G2_rev (#0052) to generate modified ARS305 locus with RS LEXA sites spanning -3 and +3 nucleosome |
| #0074 | GAAAAGGGCATGTAATATTG | Primer used for Gibson assembly PCR together with ARS316-3+3_G1pBl_rev (#0075) to generate modified ARS316 locus with RS LEXA sites spanning -3 and +3 nucleosome |
| #0075 | CTTGGGGAAGAAGTAACAATGAC | Primer used for Gibson assembly PCR together with ARS316-3+3_G1pBl_fwd (#0074) to generate modified ARS316 locus with RS LEXA sites spanning -3 and +3 nucleosome |
| #0080 | tcctccagtgggattgctacttcttttgttgctgctgcatcctcaacttg tcgatccgatgataagctgtc | Primer used for PCR together with ARS316_URA3 rev (#0081) to amplify URA3 marker gene with homology arms for genomic deletion of ARS316 |
| #0081 | gcctagcgggctattaccttgtaaataccacactatcaatccttaaatgt tgccacctgacgtctaagaa | Primer used for PCR together with ARS316_URA3 fwd (#0080) to amplify URA3 marker gene with homology arms for genomic deletion of ARS316 |
| #0119 | aaatcgtggtcgaccggcatgcaagctcccTCGAGGACAGACCACTTATG | Primer used for Gibson assembly PCR together with ARS305_2_gib_rev (#0120) to generate pM49.2 with ARS305 +-2 nucleosome |
| #0120 | aaccagtggttatatgtacagtagtacgttATCTCTCCGCCTGAATAAG | Primer used for Gibson assembly PCR together with ARS305_2_gib_fwd (#0119) to generate pM49.2 with ARS305 +-2 nucleosome |
| #0121 | aaatcgtggtcgaccggcatgcaagctcccTCGAGAAATACAGAATAGGAAAG | Primer used for Gibson assembly PCR together with ARS305_3_gib_rev (#0122) to generate pM49.2 with ARS305 +-3 nucleosome |
| #0122 | aaccagtggttatatgtacagtagtacgttATCGTAGCGGTGTTTATC | Primer used for Gibson assembly PCR together with ARS305_3_gib_fwd (#0121) to generate pM49.2 with ARS305 +-3 nucleosome |
| #0137 | TTTTCGCTGCTTGTCCTTTT | qPCR detection of the K71 plasmid spike-in for the affinity purifications (Affinity purification) |
| #0138 | CATTTTCGTCCTCCCAACAT | qPCR detection of the K71 plasmid spike-in for the affinity purifications (Affinity purification) |
| #0223 | Ggcttttcgatcagacttggcatgtgactaatcaagtatggcatgctggt ttttgggtcctttgttttcg | Primer used together with 0224 to amplify ARS locus from K111 – K113 with 50 additional homologue nts for trafo into Y10 |
| #0224 | Tagtaaataacggagactggcgaaccgaatgggcacctgcctctgactgc atttggagggaggagaagga | Primer used together with 0223 to amplify ARS locus from K111 – K113 with 50 additional homologue nts for trafo into Y10 |
| #0251 | TAACTTCAGCACCAAAGCCAACAACTACGACCTATGTCGAGCAACGACTTTCCTCCAGTGGGATTGCTAC | Primer used together with 0252 to amplify ARS locus from K114 – K116 with 50 additional homologue nts for trafo into Y11 |
| #0252 | TTCTTGGCAGTCACATATATGGAAGGTGAATTTAGAGTAGTTTCCTTATAGCCTAGCGGGCTATTACCTT | Primer used together with 0251 to amplify ARS locus from K114 – K116 with 50 additional homologue nts for trafo into Y11 |
| #0270 | TCAgctagcttaattaaAAGACAACAGATTTATTGTA | Primer used together with 0271 for 1st cloning step to get lexA/RS expression vector |
| #0271 | TCAggcgcgccCCCGAGGATTATAATTGTTC | Primer used together with for 1st cloning step to get lexA/RS expression vector |
| #0272 | tcaGGCGCGCCtttcgtctcgcgcgtttcgg | Primer used together with 0273 to amplify insert from K9 for 1st cloning step to get lexA/RS expression vector |
| #0273 | tcaGCTAGCagtagttggaatatcataat | Primer used together with 0272 to amplify insert from K9 for 1st cloning step to get lexA/RS expression vector |
| #0274 | tcaGCTAGCgagaatttgtattttcaggg | Primer used together with 0275 to amplify insert from K9 for 2nd cloning step to get lexA/RS expression vector |
| #0275 | tcaTTAATTAAccccgttccacaacacaaca | Primer used together with 0274 to amplify insert from K9 for 2nd cloning step to get lexA/RS expression vector |
| #0319 | AAC GAATTC aaaaaccccagcagcagata | Primer used for PCR together with ARS313_downstream (#0320) to amplify ARS313 replication origin in pBlueSkript using HindIII and EcoRI site adapter |
| #0320 | AAC AAGCTT ctcctgggccttgatgatac | Primer used for PCR together with ARS313_upstream (#0319) to amplify ARS313 replication origin in pBlueSkript using HindIII and EcoRI site adapter |
| #0321 | gtggtcgaccggcatgcaagctccctttcctgggcggtttggttac | Primer used for Gibson assembly PCR together with ARS313+-1_rev (#0322) to generate modified ARS313 locus with RS LEXA sites spanning -1 and +1 nucleosome |
| #0322 | gtggttatatgtacagtagtacgttctaactgtaggcgcttttatctcc | Primer used for Gibson assembly PCR together with ARS313+-1_fwd (#0321) to generate modified ARS313 locus with RS LEXA sites spanning -1 and +1 nucleosome |
| #0323 | AAC GAATTC GGGAAGGAGATCTGCAGTGT | Primer used for PCR together with ARS315_downstream (#0324) to amplify ARS315 replication origin in pBlueSkript using HindIII and EcoRI site adapter |
| #0324 | AAC AAGCTT CGATTGAAGCCGTGGATGAG | Primer used for PCR together with ARS313_upstream (#0323) to amplify ARS313 replication origin in pBlueSkript using HindIII and EcoRI site adapter |
| #0330 | gtggttatatgtacagtagtacgttGGTTGGGGTATTAAGAGTAC | Primer used for Gibson assembly PCR together with ARS315+-3_fwd (#0329) to generate modified ARS315 locus with RS LEXA sites spanning -1 and +1 nucleosome |
| #0331 | gtggtcgaccggcatgcaagctcccTTTTGTATGAAAAACTCATGAATC | Primer used for Gibson assembly PCR together with ARS313+-2_rev (#0332) to generate modified ARS313 locus with RS LEXA sites spanning -1 and +1 nucleosome |
| #0332 | gtggttatatgtacagtagtacgttCTTGGTGCAAGAAGTTGG | Primer used for Gibson assembly PCR together with ARS313+-2_fwd (#0331) to generate modified ARS313 locus with RS LEXA sites spanning -1 and +1 nucleosome |
| #0333 | gtggtcgaccggcatgcaagctcccGCCATGCCTAAGAAAGATTG | Primer used for Gibson assembly PCR together with ARS313+-3_rev (#0334) to generate modified ARS313 locus with RS LEXA sites spanning -1 and +1 nucleosome |
| #0334 | gtggttatatgtacagtagtacgttAGGCAGTTTCATCTTCAG | Primer used for Gibson assembly PCR together with ARS313+-3_rev (#0333) to generate modified ARS313 locus with RS LEXA sites spanning -1 and +1 nucleosome |
| #0338 | Agaaagtgcttttggatcgtccggtgaaattgcagtaataccgatagtcc tcgatccgatgataagctgtc | Primer used for PCR together with ARS313_URA3 rev (#0339) to amplify URA3 marker gene with homology arms for genomic deletion of ARS313 |
| #0339 | Tctacaatcaatatcctattttggccaccagctttctcttcaattagtat tgccacctgacgtctaagaa | Primer used for PCR together with ARS313_URA3 fwd (#0338) to amplify URA3 marker gene with homology arms for genomic deletion of ARS313 |
| #0346 | gaccgataaaaagggtaatagatggCCACGATTTGATGAAAGAATAAC | Primer used together with ARS313_pBlueSk_RS+-1_rev (#347) to generate the plasmid pBlueskript with ARS313+-1 plus RS+LexA |
| #0347 | ttgatactacaggagctggagatacTCGACCCGAGATCATATC | Primer used together with ARS313_pBlueSk_RS+-1_fwd (#346) to generate the plasmid pBlueskript with ARS313+-1 plus RS+LexA |
| #0350 | tcagcaatcatcattattccCCACGATTTGATGAAAGAATAAC | Primer used together with ARS313_pBlueSk_RS+-2_rev (#351) to generate the plasmid pBlueskript with ARS313+-2 plus RS+LexA |
| #0351 | atatcgtaaaatttgtgagcTCGACCCGAGATCATATC | Primer used together with ARS313_pBlueSk_RS+-2_fd (#350) to generate the plasmid pBlueskript with ARS313+-2 plus RS+LexA |
| #0366 | gggtggtatagcccttggtaCCACGATTTGATGAAAGAATAAC | Primer used together with ARS313_pBlueSk_RS+-3_rev (#367) to generate the plasmid pBlueskript with ARS313+-3 plus RS+LexA |
| #0367 | ataacccctcacctttcaagTCGACCCGAGATCATATC | Primer used together with ARS313_pBlueSk_RS+-3_fd (#366) to generate the plasmid pBlueskript with ARS313+-3 plus RS+LexA |
| #0368 | CTTGAAAGGTGAGGGGTTATATAC | Primer used together with psk_ars313+-3_fd (369) to generate the plasmid pm49.2 with ars313+-3 |
| #0369 | TACCAAGGGCTATACCAC | Primer used together with psk_ars313+-3_fd (368) to generate the plasmid pm49.2 with ars313+-3 |
| #0457 | atactaattgaagagaaagctggtggccaaaataggatattgattgtaga tgccacctgacgtctaagaa | Primer used for PCR together with ARS313_URA3 fwd (#0338) to amplify URA3 marker gene with homology arms for genomic deletion of ARS313 |
| #0458 | Gagcttttctttcctctctcttttttttttcttgttacatattcctatat tgccacctgacgtctaagaa | Primer used for PCR together with ARS315_URA3 fwd (#0340) to amplify URA3 marker gene with homology arms for genomic deletion of ARS315 |
| #0463 | TTTCATGTACTGTCCGGTGT | qPCR detection of ARS305 (Affinity purification, Replication timing) |
| #0466 | TTTTTAGCCCCCGTGTAAGTT | qPCR detection of ARS305 (Affinity purification, Replication timing) |
| #0551 | AGTCGTCCTTTATCTTCTCCCT | Primer used together with 697 to design a probe for ARS313+-3 locus for REA |
| #0552 | TCAAAGAAAAGGTGCTGCTGA | qPCR detection of ARS313 (Affinity Purification, Replication timing) and Primer used to design a probe for ARS313 next to BsaI together with 553 (test recombination) |
| #0553 | TCTTCCGTCTTAAAAGGTAGCAC | qPCR detection of ARS313 (Affinity Purification, Replication timing) and Primer used to design a probe for ARS313 next to BsaI together with 552 (test recombination) |
| #0697 | TCATCTTCAGGCTTGTTGGT | Primer used together with 697 to design a probe for ARS313+-3 locus for REA |
| #0698 | AGTGCTTGTAACTGGTGCTTG | Primer used to design a probe together with #699 on ARS305 for REA |
| #0699 | ACTGTCATAAGTGGTCTGTCGA | Primer used to design a probe together with #698 on ARS305 for REA |
| #0769 | tacgccaacttaagaccatg | Primer used to amplify Tef/ADH promotor region |
| #0770 | tctcttttccatggtcatga | Primer used to amplify Tef/ADH promotor region |
| #0775 | gtggtcgaccggcatgcaagctcccaaaaaaccgcgaagagctcc | Primer used to amplify the pBlueSkript ARS315 +-2 together with 776 in order to use th PCR fragment for Gibson assemply with pM49.2ARS315 backbone |
| #0776 | gtggttatatgtacagtagtacgttctcaaccgcagaacccgg | Primer used to amplify the pBlueSkript ARS315+-2 together with 775 in order to use the PCR fragment for Gibson assemply with pM49.2ARS315 backbone |
| #0777 | gtggtcgaccggcatgcaagctcccggttggggtattaagagtacaatgc | Primer used to amplify the pBlueSkript ARS315+-3 together with 778 in order to use th PCR fragment for Gibson assemply with pM49.2ARS315 backbone |
| #0778 | gtggttatatgtacagtagtacgttcgcaattttcttgaacggttttttc | Primer used to amplify the pBlueSkript ARS315+-3 together with 777 in order to use th PCR fragment for Gibson assemply with pM49.2ARS315 backbone |
| #0815 | aaaaaatccggaacaaaaaaaccgcTCGACCCGAGATCATATC | Primer used to amplify the pM49.2ARS315+-2 together with 816 in order later to be used for gibson assembly with PCR fragment of K174 as template |
| #0816 | ACCAACTAATTACTGCTCAACCGCACCACGATTTGATGAAAGAATAAC | Primer used to amplify the pm49.2ars315+-2 together with 815 in order later to be used for gibson assembly with pcr fragment of k174 as template |
| #0817 | acgttattctttcatcaaatcgtggTGCGGTTGAGCAGTAATTAG | Primer used to amplify the k174 together with 818 in order later to be used for gibson assembly with PCR fragment of pM49.2ARS315+-2 as template |
| #0818 | ccacagtgatatgatctcgggtcgaGCGGTTTTTTTGTTCCGG | Primer used to amplify the k174 together with 817 in order later to be used for gibson assembly with PCR fragment of pM49.2ARS315+-2 as template |
| #0819 | actctcactattttgttttttcgacccgagatcatatc | Primer used to amplify the pM49.2ARS315+-3 together with 820 in order later to be used for gibson assembly with PCR fragment of K174 as template |
| #0820 | cgtatatgactgcacaagacccacgatttgatgaaagaataac | Primer used to amplify the pM49.2ARS315+-3 together with 819 in order later to be used for gibson assembly with PCR fragment of K174 as template |
| #0821 | gtcttgtgcagtcatatac | Primer used to amplify the k174 together with 822 in order later to be used for gibson assembly with PCR fragment of pM49.2ARS315+-2 as template |
| #0822 | aaaaacaaaatagtgagagtaatg | Primer used to amplify the k174 together with 821 in order later to be used for gibson assembly with PCR fragment of pM49.2ARS315+-2 as template |
| #0834 | ACGGCGTAATGGATCAGAAATA | Primer used together with 835 to amplify the late replicating region on Chr4 for replication timing experiments |
| #0835 | CTGGCTCACCAGAATCTTCAT | Primer used together with 834 to amplify the late replicating region on Chr4 for replication timing experiments |
| #0837 | CGGCATTATCGTACACAACCT | qPCR detection of ARS316 (Affinity purification, Replication timing) |
| #0838 | GTTCTTCGTTGCCTACATTTTCT | qPCR detection of ARS316 (Affinity purification, Replication timing) |
| #0848 | AATCTCACTAAAAGTAACATACAGTACCGATAAATCGAGATTGCAGAGTA tgccacctgacgtctaagaa | Primer used together with 849 for homologous recombination and replacement of isw2 with Ura |
| #0849 | GTTCAATTATCTTAGAATGGATATGAATTAGTTAAAGCGGCTCGACCCAG tcgatccgatgataagctgtc | Primer used together with 848 for homologous recombination and replacement of isw2 with Ura |
| #0858 | TCCATGTCCATGTCCATGTCATCATGGGCCGTGACAAGCGTCGCCGCGCA gccgaataaacttaaaattga | Primer used (together with 859) for homologous recombination between ARS315pBlueSk+RS+LexA and yeast genome having as adaptor the 50bl homologous arms |
| #0859 | CCTCGACGGCCTCCAGTTCTTCGACCAACTGTTCGTGATCGTCATCCATT gagcttttctttcctctctct | Primer used (together with 858) for homologous recombination between ARS315pBlueSk+RS+LexA and yeast genome having as adaptor the 50bl homologous arms |
| #0932 | attcaaatacgtcatcctat | PCR with K207 as template to make fragment for bar1 ko in Y8 |
| #0933 | actggatacttggtcgtcgt | PCR with K207 as template to make fragment for bar1 ko in Y0008 |
| #0944 | TGTGTGTTTGTATACTCTGTGGG | Primer used for designing a probe (together with 945) to test recombination of ars315 origin |
| #0945 | TAAGCGGCAAAACACTTGTGT | Primer used for designing a probe (together with 944) to test recombination of ars315 origin |
| #1027 | TTTCAAAACTTTCAGCCCAATCA | Primer used together with 1028 to design a probe for ARS316+-3 locus for REA |
| #1028 | CTGTGAAGACGTAATGGTGTGT | Primer used together with 1027 to design a probe for ARS316+-3 locus for REA |
| #1035 | ccctgcttattcaggcggag | Primer used for qPCR with 1036 to amplify a 150bp region 175bp upstream the origin ARS315 for REA |
| #1036 | ggtccaggtactaccatgtt | Primer used for qPCR with 1035 to amplify a 150bp region 175bp upstream the origin ARS315 for REA |
| #1049 | gggtaccgggccccccctcgaggtcgacggtatcgataagcttgatatcgcctgcaggagaaaaaaagaatagtaataacaatttg | Primer used for gibson assembly to amplify ies6 together with 1050 |
| #1050 | acattatatgacccttctagacacttgtttcgactttctcacg | Primer used for gibson assembly to amplify ies6 together with 1049 |
| #1051 | tcatcagcgtgagaaagtcgaaacaagtgtctagaagggtcatataatg | Primer used for gibson assembly to amplify histidine together with 1052 |
| #1052 | aaatacatacatacatatacaatgcaatcaaaattgtattccacgtc | Primer used for gibson assembly to amplify histidine together with 1051 |
| #1053 | ggtgacgtggaatacaattttgattgcattgtatatgtatgtatgtattttag | Primer used for gibson assembly to amplify ies6 together with 1054 |
| #1054 | ccaccgcggtggcggccgctctagaactagtggatcccccgggctgcagggagctcgttctcccaatgaatggtc | Primer used for gibson assembly to amplify ies6 together with 1053 |
| #1158 | ccctacaccacagcatacaaaaccgaatcaaaattgtattccacgtc | Primer used for pcr to amplify KanMX with homologous recombination arms for ies6 deletion together with 1159 |
| #1159 | ggtgacgtggaatacaattttgattcggttttgtatgctgtgg | Primer used for pcr to amplify KanMX with homologous recombination arms for ies6 deletion together with 1158 |
| #1299 | CAGACAAAACAGCAACGGAAC | Primer used together with 1300 for qPCR for late replicating region on chrV |
| #1300 | CTATATGGAAGACGTTGGGAAGG | Primer used together with 1299 for qPCR for late replicating region on chrV |
| #1301 | ATCCTGCGCGTTGACATAA | Primer used together with 1302 for qPCR for late replicating region on chrXIV |
| #1302 | AGATTCCGTTGCTGGCTATC | Primer used together with 1301 for qPCR for late replicating region on chrXIV |

**Supplementary Table S3: Plasmids used in this study.**

| **ID** | **Application/Purpose** | **Cloning Strategy** |
| --- | --- | --- |
| K004 (pM49.2) |  | Plasmid pM49.2 is a derivative of pABX22, and has been modified by addition of a LexA-binding cluster juxtaposed to an RS element. |
| K005 | Yeast expression vector for constitutive expression of LexA-TAP under control of TEF2 promoter and inducible expression of R Recombinase under control of GAL1-10 promoter; LEU2 selection marker framed with RS sites |  |
| K009 | E. coli/yeast shuttle vector used for genomic integration of CYC1 LexATAP GAL1-10 RecR expression cassette by recombination in URA3 locus |  |
| K018 |  | pBlueSkript SK (+) |
| K094 | Plasmid containing wildtype ARS305 locus in pBlueScript backbone | HindIII/PstI cut amplicon (primer 0016/0017) from yeast gDNA into K18 |
| K95 | Plasmid containing wildtype ARS316 locus in pBlueScript backbone | EcoRI/PstI cut amplicon (primer 0020/0021) from yeast gDNA into K18 |
| K102 | Vector with ARS305 +-2 nucleosome sequence next to lexA/RS sites | Gibson assembly of two fragments (primer 0119/0120 with K94 as template + HpaI/XhoI cut backbone from K0004) |
| K103 | Vector with ARS305 +-3 nucleosome sequence next to lexA/RS sites | Gibson assembly of two fragments (primer 0121/0122 with K94 as template + HpaI/XhoI cut backbone from K0004) |
| K112 | Vector for yeast transformation in order to modifiy ARS305 locus and insert RS sites next to NS+-2 | Gibson assembly of two fragments (primer 0046/0047 with K94 as template + primer 0048/0049 with K102 as template) |
| K113 | Vector for yeast transformation in order to modifiy ARS305 locus and insert RS sites next to NS+-3 | Gibson assembly of two fragments (primer 0050/0051 with K94 as template + primer 0052/0053 with K103 as template) |
| K116 | Vector for yeast transformation in order to modifiy ARS316 locus and insert RS sites next to NS+-3 | Gibson assembly of two fragments (primer 0074/0075 with K95 as template + primer 0076/0077 with K106 as template) |
| K139 | E. coli/yeast shuttle vector used for genomic integration of pCYC1 LexA-TAP GAL1-10 RecR expression cassette by recombination in 500bp homology region from K121 of yeast chromosome I, LEU2 selection marker framed with RS sites (two mutations in the lexA gene that stop binding to lexA binding site: V11A, N171D) | AscI/NheI cut amplicon (primer 0270/0271 template K121) inserted with AscI/NheI cut amplicon (primer 0272/0273 template K009). Resulting plasmid was cut with NheI/PacI and inserted with NheI/PacI cut amplicon (primer 0274/0275 template K009). |
| K173 | Plasmid containing wildtype ARS313 locus in pBlueSkript backbone | EcoRI/HindIII cut amplicon primers 319and 320 from gDNA into K18 |
| pM49.2_ARS313+-1 | Vector with ARS313+-1 nucleosome sequence next to lexA/RS sites | Gibson assembly of two fragments primers 321and 322 with K173 as template and HpaI/XhoI cut backbone from K004 |
| pM49.2_ARS313+-2 | Vector with ARS313+-2 nucleosome sequence next to lexA/RS sites | Gibson assembly of two fragments primers 331and 332 with K173 as template and HpaI/XhoI cut backbone from K004 |
| pM49.2_ARS313+-3 | Vector with ARS313+-3 nucleosome sequence next to lexA/RS sites | Gibson assembly of two fragments primers 333and 334 with K173 as template and HpaI/XhoI cut backbone from K004 |
| K168 | Vector for yeast transformation in order to modify ARS313 locus and insert the RS sites next to NS+-1 | Gibson assembly of two fragments primers 346and 347 with a vector of ARS313+-1 nucleosome sequence next to lexA/RS site as template and primers 348 and 349 with K173 as template |
| K167 | E. coli/yeast shuttle vector used for genomic integration of pCYC1 LexA-TAP GAL1-10 RecR expression cassette by recombination in 500bp homology region from K121 of yeast chromosome I, LEU2 selection marker framed with RS sites | Insert from K009 (NsiI/BlpI) cloned into K139 |
| K169 | Vector for yeast transformation in order to modify ARS313 locus and insert the RS sites next to NS+-2 | Gibson assembly of two fragments primers 350and 351 with a vector of ARS313+-2 nucleosome sequence next to lexA/RS sites as template and primers 352 and 353 with K173 as template |
| K170 | Vector for yeast transformation in order to modify ARS313 locus and insert the RS sites next to NS+-3 | Gibson assembly of two fragments primers 366and 367 with a vector with ARS313+-3 nucleosome sequence next to lexA/RS sites as template and primers 368 and 369 with K173 as template |
| K174 | Plasmid containing wildtype ARS315 locus in pBlueSkript backbone | EcoRI/HindIII cut amplicon primers 323and 324 from gDNA into K18 |
| K238 | E.coli/yeast shuttle vector used for genomic integration of pTEF2 LexA-TAP GAL1-10 RecR expression cassette by recombination in 500bp homology region from K121 of yeast chromosome I, LEU2 selection marker framed with RS sites | NcoI/AflII cut amplicon (primer 0769/0770 template K005) into K167 |
| K293 | Vector for yeast transformation in order to modify ARS315 locus and insert the RS sites next to NS+-2 | Gibson assembly of two fragments primers 815and 816 with a vector of ARS315+-2 nucleosome sequence next to lexA/RS sites as template and primers 817 and 818 with K174 as template |
| K273 | Vector for yeast transformation in order to modify ARS315 locus and insert the RS sites next to NS+-3 | Gibson assembly of two fragments: primers 819 and 820 with a vector with ARS315+-3 nucleosome sequence next to lexA/RS sites as template and primers 821 and 822 with K174 as template |
| K207 |  | Bar1 knock-out plasmid |
| K303 | Vector for yeast transformation in order to delete the Ies6 gene and replace it with histidine | Gibson assembly of four fragments: Primers 1049 and 1050 , 1053 and 1054 with gDNA as template, Primers 1051 and 1052 with K207 plasmid as template, K18 digested by |
